# Small RNA pathways and diversity in model legumes: lessons from genomics

**DOI:** 10.3389/fpls.2013.00236

**Published:** 2013-07-10

**Authors:** Pilar Bustos-Sanmamed, Jérémie Bazin, Caroline Hartmann, Martin Crespi, Christine Lelandais-Brière

**Affiliations:** ^1^Centre National de la Recherche Scientifique, Institut des Sciences du VégétalGif-sur-Yvette Cedex, France; ^2^Université Paris Diderot, U.F.R. Sciences du VivantParis Cedex 13, France

**Keywords:** small RNA, dicer, argonaute, model legumes

## Abstract

Small non-coding RNAs (smRNA) participate in the regulation of development, cell differentiation, adaptation to environmental constraints and defense responses in plants. They negatively regulate gene expression by degrading specific mRNA targets, repressing their translation or modifying chromatin conformation through homologous interaction with target loci. MicroRNAs (miRNA) and short-interfering RNAs (siRNA) are generated from long double stranded RNA (dsRNA) that are cleaved into 20–24-nucleotide dsRNAs by RNase III proteins called DICERs (DCL). One strand of the duplex is then loaded onto effective complexes containing different ARGONAUTE (AGO) proteins. In this review, we explored smRNA diversity in model legumes and compiled available data from miRBAse, the miRNA database, and from 22 reports of smRNA deep sequencing or miRNA identification genome-wide in three legumes: *Medicago truncatula*, soybean (*Glycine max*) and *Lotus japonicus*. In addition to conserved miRNAs present in other plant species, 229, 179, and 35 novel miRNA families were identified respectively in these 3 legumes, among which several seems legume-specific. New potential functions of several miRNAs in the legume-specific nodulation process are discussed. Furthermore, a new category of siRNA, the phased siRNAs, which seems to mainly regulate disease-resistance genes, was recently discovered in legumes. Despite that the genome sequence of model legumes are not yet fully completed, further analysis was performed by database mining of gene families and protein characteristics of DCLs and AGOs in these genomes. Although most components of the smRNA pathways are conserved, identifiable homologs of key smRNA players from non-legumes, like AGO10 or DCL4, could not yet be detected in *M. truncatula* available genomic and expressed sequence (EST) databases. In contrast to *Arabidopsis*, an important gene diversification was observed in the three legume models (for DCL2, AGO4, AGO2, and AGO10) or specifically in soybean for DCL1 and DCL4. Functional significance of these variant isoforms may reflect peculiarities of smRNA biogenesis and functions in legumes.

## Introduction

Small RNAs (smRNAs) are important riboregulators in bacteria, fungi, plants and animals, which negatively regulate the expression of specific target genes by base-pairing. In the last decade, smRNA functions have been largely described in plants, both in development and responses to biotic and abiotic interactions (for review, Khraiwesh et al., 2012). Plant smRNAs, 20–24 nucleotides (nt) in length, are classically divided into microRNAs (miRNAs) and short-interfering RNAs (siRNAs). Like miRNAs, the natural-antisense siRNAs (natsiRNA) and the trans-acting siRNAs (tasiRNAs) are two classes of siRNAs involved in post-transcriptional gene regulation by degrading and/or inhibiting translation of their mRNA targets. In contrast, the heterochromatin-associated siRNAs (hcsiRNA) are associated to chromatin modifications and transcriptional repression of their target DNA loci.

**Figure 1:**
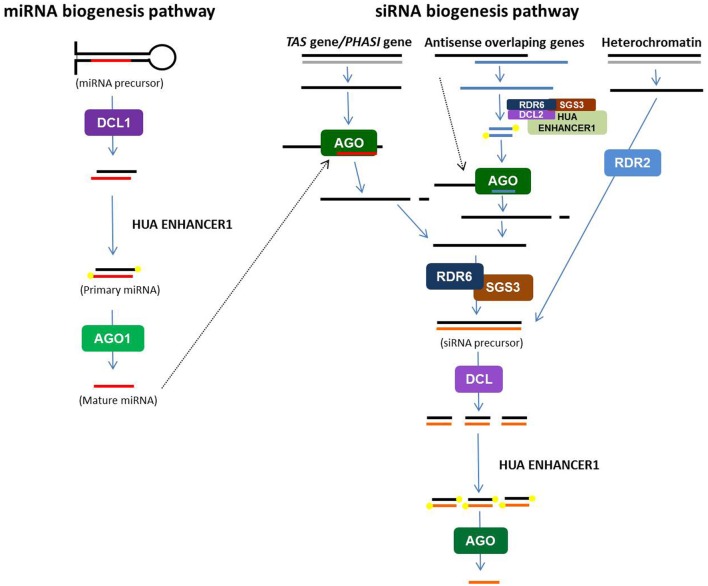
**Schematic representation of microRNA and short-interfering RNA pathways in plants**. Double stranded RNA (dsRNA) precursors are cleaved by specific Dicer-like (DCL) proteins to produced small dsRNA duplexes. After 3′OH methylation by HUA ENHANCER1, one strand of duplex is loaded onto silencing effector complexes (RISC/RITS) that contain ARGONAUTE (AGO) proteins. According the category of small RNA, the origin of the dsRNA precursor and the DCL and AGO proteins involved are different (see text).

All plant smRNAs are generated from long double stranded RNA (dsRNA) precursors that are cleaved by RNase III proteins, called DICER-like (DCLs). However, miRNAs and siRNAs derive from different types of precursors (Figure 1; Voinnet, 2009). The result of DCL action is the production of 20–24-base-pair (bp) RNA duplexes with 2 nt long 3′ overhangs (Vazquez, 2006). After stabilization by 3′OH methylation (Bove et al., 2006), one strand of the smRNA duplex is loaded onto a silencing effector complex (called RISC/RITS), that contains one ARGONAUTE (AGO) protein, to mediate gene silencing by base pairing with their targets (Vazquez, 2006; Vaucheret, 2008; Mallory and Vaucheret, 2010). In *Arabidopsis thaliana*, 4 DCL and 10 AGO genes have been described and play different roles in smRNA biogenesis and action.

MiRNAs, mainly 21–22 nt in length, are involved in the post-transcriptional regulation of gene expression. Transcribed by the RNA polymerase II from specific genes, the miRNA primary transcripts have an imperfect dsRNA stem-loop structure that is processed by DCL1 into a miR/miR^*^ duplex. After stabilization by 3′-O-methylation and transport to the cytoplasm, the mature miRNA is loaded into a RISC complex through the AGO1 or AGO10 proteins (Brodersen et al., 2008). Inside this complex, the miRNA will bind a complementary target RNA, leading to inhibition of its translation or degradation/destabilization (Voinnet, 2009). In plants, a set of approximately 20 conserved miRNA families, first identified in *Arabidopsis thaliana*, rice or poplar, have been identified in almost all angiosperms studied (Cuperus et al., 2011). Most conserved families are composed of several genes, that code either for a unique mature miRNA or for different but very similar variants. Their targets are also generally conserved among plants (Allen et al., 2004). In contrast, species- or lineage-specific miRNAs are generally present in low amount and encoded by unique genes or small gene families (Cuperus et al., 2011; Turner et al., 2012).

NatsiRNAs arise from natural cis- or trans- antisense overlapping transcripts. One transcript is usually constitutively expressed, while the second is under the control of an promoter responding either to abiotic stresses or pathogen attack (reviewed in Khraiwesh et al., 2012). Their biogenesis seems quite complex and involved successively DCL2 and DCL1 to produce an original natsiRNA of 22 nt and several secondary 21 nt natsiRNAs (for a more detail description, Zhang et al., 2012).

TasiRNAs are generated from transcripts of non-protein coding genes, called *TAS*, The *TAS* transcripts are first cleaved through the action of specific miRNAs through AGO1 or AGO7. The resulting *TAS* cleavage products are transcribed by RDR6, a RNA dependent RNA polymerase, into long dsRNAs, which are processed by DCL4 into several different 21 nt tasiRNAs following a 21 nt phased interval (Vaucheret, 2005; Voinnet, 2009; Allen and Howell, 2010). Some tasiRNAs can function in a similar way to miRNAs and regulate genes *in trans* different from their precursors. In *A. thaliana*, to date, four TAS genes have been intensively studied (Allen et al., 2005). For instance, *TAS2*-tasiRNAs negatively regulate genes of pentatricopeptide repeat family (PPRs); *TAS3*-tasiRNAs target transcription factors (TF) of the Auxin Response Factor (ARF) family and *TAS4*-tasiRNAs regulate MYB TFs involved in the biosynthesis of anthocyanins.

The last category of siRNA, called hcsiRNAs, are 24 nt in size and mainly derive from heterochromatic DNA regions, transposable elements, regions surrounding centromeres and repetitive sequences. The RNA dependent RNA polymerase RDR2 uses long ssRNA transcribed from heterochromatic regions by the polymerase IV (Onodera et al., 2005), as templates to produce dsRNAs which are processed by DCL3 into several 24 nt hc-siRNAs (Lu et al., 2006). Loaded into a RIST complex containing AGO4, AGO6, or AGO9 proteins, these siRNAs bind complementary DNA loci and induce their methylation through RNA-mediated DNA methylation (RdDM, Havecker et al., 2010; Olmedo-Monfil et al., 2010).

As plant smRNAs play key roles in several developmental stages and in responses to stress, the question of their involvement in the symbiotic nodulation process in legumes has rapidly been addressed by the scientific community. To our knowledge, the first miRNA reported to regulate nodule development (Combier et al., 2006), was miR169, which acts as a negative regulator of *HAP2*. Repression of *HAP2*, which belongs to the CCAAT binding TF family, decreased the number of nodules and altered nodule morphology in the model legume *Medicago truncatula*. Plants that over-expressed one mt-tmiR169 precursor showed the same phenotype as plants where *HAP2* was inactivated by RNA interference. Combier et al. (2006) proposed that restriction of spatial and temporal expression of the *MtHAP2* target in specific nodule regions was tightly regulated by this miRNA. Afterwards, other miRNAs were associated to nodulation in legumes as reviewed elsewhere (Simon et al., 2009; Khan et al., 2011; Bazin et al., 2012). In parallel, during the five last years, rapid progress in sequencing technologies (from 454 pyrosequencing to SOLEXA and SOLID) allowed the legume scientific community first, to sequence the genomes and second, to characterize genome-wide a large set of smRNAs from the three legumes: *Medicago truncatula, Lotus japonicus* and *Glycine max*. Even though genome sequences of three model legumes are not yet completely finished, a very large portion is available in genomic and EST databases to permit gene identification and annotation. However, we cannot exclude that the lack of certain sequences in these databases may be due to its low expression and presence in non-sequenced regions.

In the first part of this review, we will focus on smRNA diversity in model legumes, present recent data about the miRNA functions in nodulation and define a novel category of siRNAs, the phased siRNAs, likely associated with defence reactions in legumes. In the second part, we mined public genomic databases and published reports to investigate the conservation and specificities of the main components of the smRNA pathways [DICER-like and ARGONAUTE (AGO) proteins] in the three model legumes.

## Legume miRNA diversity and functions in nodulation

### Deep sequencing revealed a large diversity of conserved and novel miRNAs

**Figure 2:**
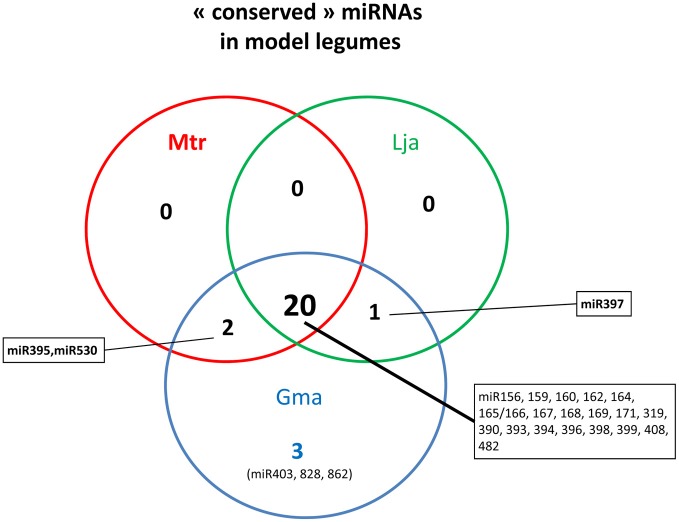
**“Conserved” miRNAs in the three model legumes**. For *Glycine max* and *Medicago truncatula*, data were obtained from miRBase (v. 19.0) and for *Lotus japonicus* from De Luis et al. (2012).

Since 2006, most miRNAs from animal and plant species have been registered in the miRNA database, called miRBAse (www.mirbase.org/, Griffiths-Jones et al., 2006). Taking into account v19.0 (August 2012) for *M. truncatula* and *G. max* and the recently identified miRNAs, from *L. japonicus* (De Luis et al., 2012), we listed 26 conserved miRNA families (Figure 2, Table 1). Most of them (from miR156 to miR399) corresponded to the set of 21 conserved miRNAs found in nearly all angiosperms (Sunkar and Jagadeeswaran, 2008). However, the absence of 6 conserved families in at least one or even two legumes was unexpected. For instance, miR395 and miR530, two miRNAs regulated by sulfur and nitrogen starvation respectively in *Arabidopsis* (Kawashima et al., 2009; Liang et al., 2010, 2012), were not reported in *L. japonicus*. Only 3 smRNA libraries have been sequenced in this species, and miRNAs having low accumulation levels in those samples may have been missed. We thus searched these miRNAs in available *L. japonicus* genomic data and identified 5 miR395 and one miR530 genes. In contrast, even after deep sequencing of a large variety of tissues and conditions, miR397 was never reported in *M. truncatula*. Lack of this miRNA was first noticed by Sunkar and Jagadeeswaran (2008), who performed an *in silico* prediction of conserved miRNAs in genomic sequences from 682 species, including the three model legumes. In agreement with those observations, no miR397 sequence was found in the last version of *M. truncatula* genome (Young et al., 2011). Putative roles of miR397 in *G. max* and *L. japonicas*, which develop determinate nodules, will be discussed later. Finally, three conserved miRNAs, first described in *A. thaliana*, were only found in *G. max* (miR403, miR828 and miR862) but not in the other legumes. Among them, miR403 is generally considered as dicot-specific and was found in 16 non-legume species. This miRNA may have been lost in certain legume lineages. The presence of miR862 in soybean was striking as, until then, this miRNA was only reported in the *Arabidopsis* genus and in tomato (Gu et al., 2010). In this latter species, miR862 exhibited differential accumulation in roots submitted to phosphate starvation and interacting with arbuscular mycorrhizal (AM) fungi. Hence, although the majority of the conserved miRNAs are present in the three model legumes, specific loss or gains of miRNA genes occurred in certain legume lineages or species.

**Table 1:**
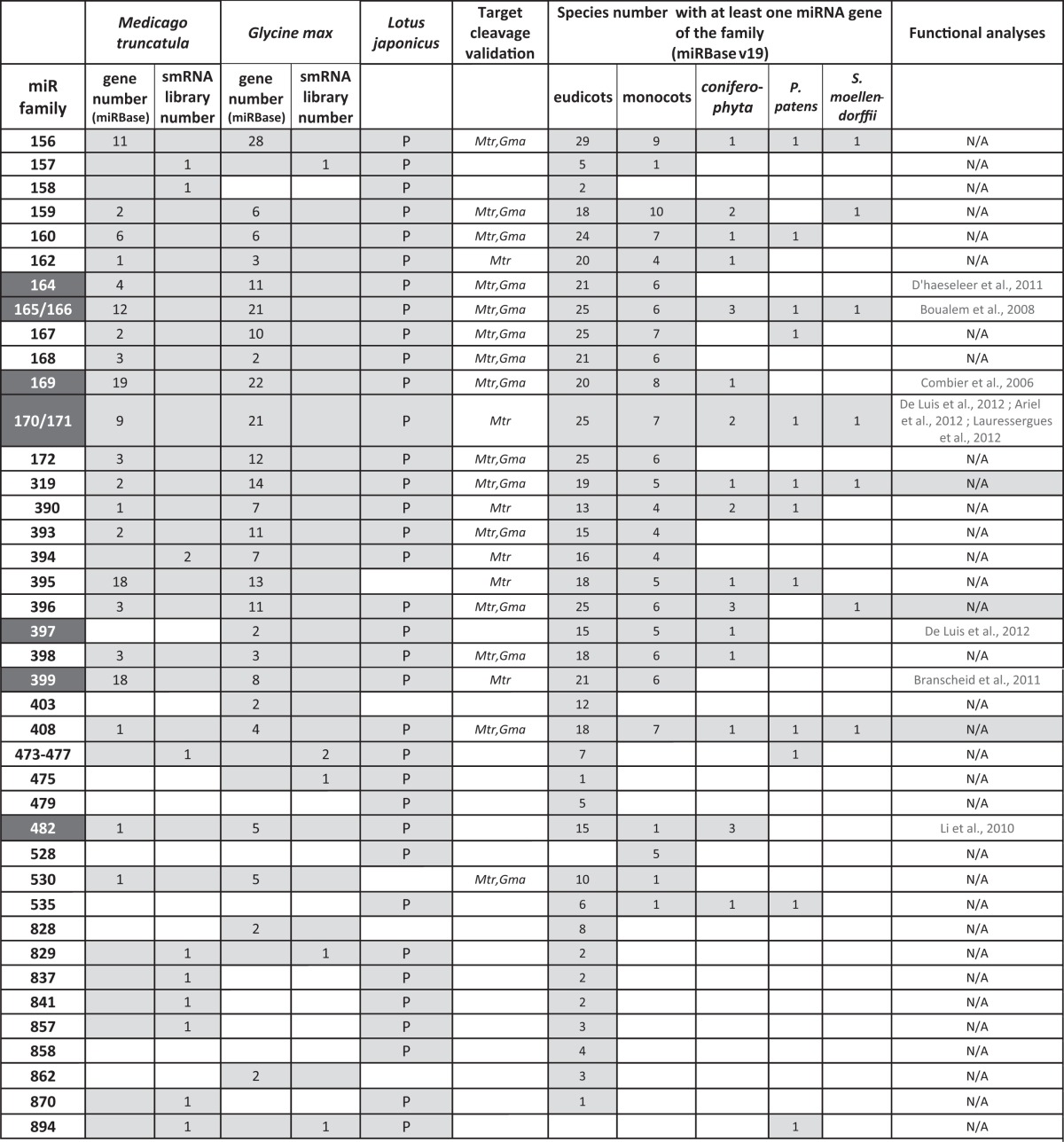
**«Conserved» miRNA families in the three model legumes**.

*For each conserved miRNA family, the number of genes listed in miRBAse v19 in Medicago truncatula and soybean (Glycine max) are given. If one miRNA was absent from miRBAse, we searched it in smRNA libraries or genome reports and indicated the number of reports where it was identified. References of the smRNA sequencing and genome reports used for these additional searches are (Jagadeeswaran et al., 2009; Kulcheski et al., 2011; Lelandais-Briere et al., 2009; Li et al., 2011; Radwan et al., 2011; Szittya et al., 2008; Turner et al., 2012; Wong et al., 2011; Young et al., 2011; Zhai et al., 2011). Presence in L. japonicus according De Luis et al. (2012) is indicated by P. Validation of target cleavage by degradome experiments in M. truncatula and/or G. max were extracted from the following reports: (Devers et al., 2011; Kulcheski et al., 2011; Song et al., 2012; Turner et al., 2012; Zhai et al., 2011; Zhou et al., 2012); N/A, not applicable. P. patens: Physcomitrella patens (moss); S. moellendorfii: Selaginella moellendorfii (lycophyta) Pale grey boxes: presence of the miRNA, dark gray boxes: miRNA with functional analyses in legumes*.

Conserved miRNAs are generally encoded by multigenic families (Allen et al., 2004). In miRBAse, gene number per miRNA family was generally higher in soybean than in *M. truncatula* (Table 1). This was expected as the large soybean genome size, estimated at ~1115 Mbp, was associated to remnants of a whole genome duplication event, which occurred approximately ~13 Mya ago in soybean (Schmutz et al., 2010). Nevertheless, the ratio between gene numbers in *G. max* and *M. truncatula* is generally above two, suggesting that genome duplication was not the only event explaining miRNA diversification in *G. max*. Surprisingly, for some families, an opposite profile was observed: for instance, miR395 and miR399 genes, generally organized in clusters, were more abundant in *M. truncatula* than in soybean (18/13 genes for miR395 and 18/8 genes for miR399 according to miRBase). However, searches of miR395 and miR399-like sequences in the *G. max* genomic database, allowing three mismatches, allowed us to identify 30 and 20 putative members respectively (C. Lelandais, pers. communication), thus suggesting that all genes of these large miRNA families in soybean have not yet been registered in miRBase.

Globally, DCL1 recognize imperfect dsRNA regions like those present in miRNA precursors to release 21–22 nt miRNAs and is the major enzyme involved in miRNA biogenesis. On the other hand, DCL4 also produces 21 nt smRNAs but only from fully complementary dsRNA. In *A. thaliana*, some miRNAs (miR822, miR839, and miR869) are processed by the action of DCL4 instead of DCL1 (Rajagopalan et al., 2006; Ben Amor et al., 2009). Those miRNAs are present in inverted repeats, share high similarity with their targets and it has been proposed that inverted duplication events formed self-complementary regions which could generate new miRNA genes (Allen et al., 2004). To date, none of these DCL4-dependent miRNAs was described in legumes, reinforcing the hypothesis of their recent and specific origin in the *Arabidopsis* genus. On the other hand, conserved miRNAs can evolve into new miRNA variants that may regulate novel targets. For instance, in *M. truncatula*, a 20 nt variant of miR156 was able to cleave a novel WD40 target in addition to the conserved Squamosa-Binding Protein TF targets (Naya et al., 2010). More recently, a novel isoform of miR171 was discovered in *M. truncatula* and *L. japonicus* (Devers et al., 2011; Bazin et al., 2012; De Luis et al., 2012), that repress a key actor of symbiotic signaling, *NSP2* (Nodulation Signaling Pathway 2, a GRAS TF). Further analyses of the miR171 family using both miRBase and comparative genomics (Bazin et al., 2012) revealed that this isoform is also present in non-legume species, such as *Populus trichocarpa* (ptc-miR171) and *Citrus sinensis* (csi-miR171b). However several plants unable to form root endomycorrhiza like *A. thaliana, Brassica napus* and the gymnosperm *Pinus taeda*, do not contain this isoform suggesting a role in root symbioses.

Several non-conserved miRNAs, first described in one legume species (soybean: Subramanian et al., 2008; Wang et al., 2009 or *M. truncatula*: Szittya et al., 2008 and Jagadeeswaran et al., 2009) have been reported as “legume-specific”. In Table 2 we summarize some characteristics of 15 of them selected either because they are present in at least two of the legumes analyzed or because they have been functionally related to nodulation (miR1512, miR1515; miR1521; Li et al., 2010). According to miRBase, all, except miR1511, miR1515, miR2111 and miR2118, may be considered as “legume-specific”. However, Zhai et al. (2011) showed that variants of miR1507 and miR1509 were present in smRNA libraries of non-leguminous species. For instance, miR1507 was highly abundant in grapes. In addition, miR2118 was sequenced in 34 non-legume species, including 4 gymnosperms, suggesting a very ancestral origin (>250 million years). During the last three years, several reports of smRNA library sequencing and genome wide miRNA identification (see Data Sheet 1 for the list) allowed to found hundreds of novel miRNA families in legumes. In miRBAse, 229 and 179 novel miRNA families have been registered for *M. truncatula* and *G. max*, respectively, whereas De Luis et al. (2012) reported 35 novel miRNA families in *L. japonicus*. In miRBAse, *M. truncatula* and *G. max* correspond to the first and third plant species in term of total numbers of miRNA genes, with 675 and 506 genes, respectively. This huge diversity, comparable to, e.g., rice, may be due to the high number of smRNA deep sequencing analyses performed (9 and 10 reports for *M. truncatula* and *G. max*, respectively, listed in Data Sheet 1). In addition, likely “false” candidates have been registered due to low stringency criteria used for miRNA identification in some of the initial studies. For instance, between 2009 and 2011, many miRNAs (including certain identified in our work, Lelandais-Briere et al., 2009), were registered in miRBAse, although no miR^*^ was sequenced in the libraries, a criteria that became required for bona fide miRNAs (Meyers et al., 2008).

**Table 2:**
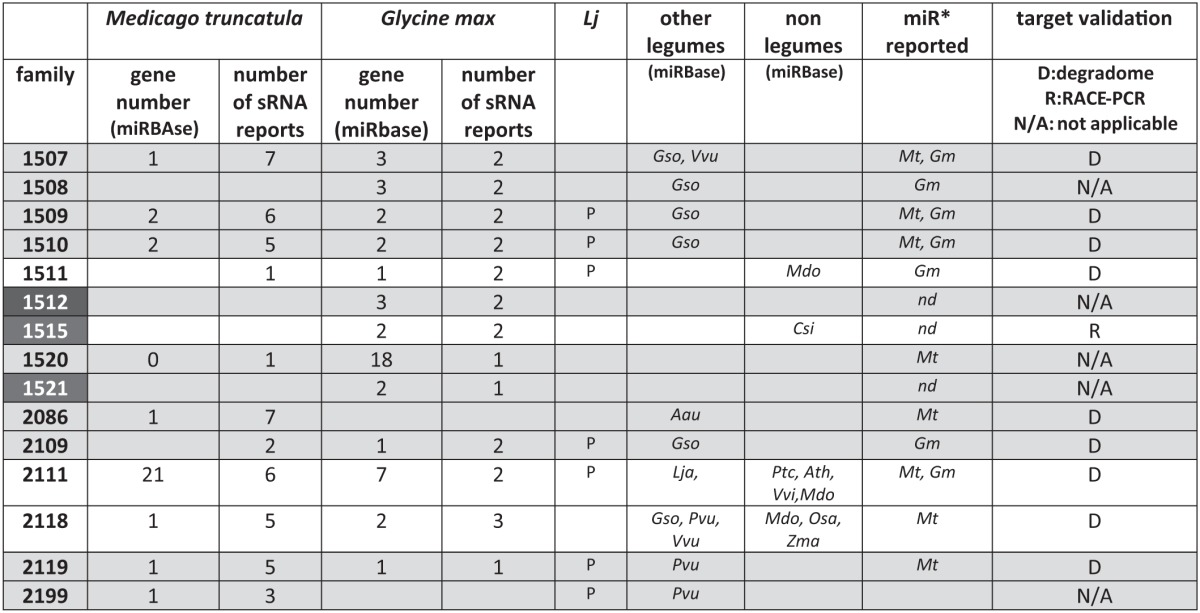
**Selection of 15 “legume” miRNA families**.

*For selected “legume” miRNA families, the number of genes listed in miRBAse v19 in Medicago truncatula and soybean (Glycine max) species are given. The number of smRNA sequencing or genome reports where it was cited is also indicated. Reports of smRNA sequencing and genome sequencing used for these additional searches are: (Jagadeeswaran et al., 2009; Kulcheski et al., 2011; Lelandais-Briere et al., 2009; Li et al., 2011; Radwan et al., 2011; Szittya et al., 2008; Turner et al., 2012; Wong et al., 2011; Young et al., 2011; Zhai et al., 2011). Presence in L. japonicus (Lj) according to De Luis et al. (2012) is indicated by P. Validation of target cleavage by degradome experiments in M. truncatula and/or G. max were extracted from the following reports (Devers et al., 2011; Kulcheski et al., 2011; Song et al., 2011; Turner et al., 2012; Zhai et al., 2011; Zhou et al., 2012). P. patens: Physcomitrella patens (moss); S. moellendorfii: Selaginella moellendorfii (lycophyta). Other Legumes species: Pvu (Phaseolus vulgaris); Vvu (Vigna unguiculata); Ahy (Arachis hypogea); Aau (Acacia auriculiformis); Gso (Glycine soja); Non legume species cited in the table: Ath (Arabidopsis thaliana); Mdo (Malus domestica); Osa (Oryza sativa); Ptc (Populus trichocarpa); Vvi (Vitis vinifera); Zma (Zea mays). Pale grey lines: legume-specific miRNAs (according miRBAse data); dark grey boxes: miRNA with functional analyses in legumes (Li et al., 2010)*.

In this context, a major challenge in the next years will be to select the “best” novel legume miRNAs for functional analyses. Although several nodulation-responsive miRNAs have already been identified (Subramanian et al., 2008; Simon et al., 2009; Li et al., 2010; De Luis et al., 2012; Turner et al., 2012), microarray based genome-wide transcriptional analyses as well as robust statistical comparisons of smRNA abundancies in libraries from various developmental stages or tissues will be very helpful. In addition to miRNA expression, it is of interest to analyse their target mRNAs. Recent analyses used “degradome” experiments (also called PARE, German et al., 2009) to detect enrichment of cleaved mRNAs on miRNA complementary sites. By analysing changes in miRNA expression patterns which correlate with specific degradation of mRNA targets, we can assess the potential regulation by miRNAs of several mRNA targets, both in *G. max* (Song et al., 2011; Turner et al., 2012) and *M. truncatula* (Devers et al., 2011; Zhai et al., 2011; Zhou et al., 2012). For instance, the mRNAs encoding a salt-tolerance protein and a xyloglucan endo-transglucosylase/hydrolase (an enzyme that participates in cell wall formation) are targeted by miR2708 and miR2687, respectively, in response to the presence of mercury (Zhou et al., 2012). In addition, mtr-miR2681 targets several transcripts coding TIR-NBS-LRR resistance proteins and cleavage of five of them (TC127116, TC115294, TC128879, BG587250, and NP7251801) were identified in roots treated with 10 μM Hg (Zhou et al., 2012). Degradome data in mycorrhized roots or control conditions showed that genes associated to defense responses (Medtr6g098880.1, Medtr2g046350.1, and TC138295) are specifically regulated by miR5213 or miR2678, respectively (Devers et al., 2011). Interestingly, miR5213 was conserved among species able to interact with AM fungi (Devers et al., 2011). Finally, this degradome allowed Devers et al. (2011) to propose that some miR^*^ (complementary smRNA to the miRNA generated by DCL processing) accumulating at high levels in *M. truncatula*, were able to cleave complementary mRNA targets. The miR169^*^ cleaved *MtBCP1* transcripts coding for an arbuscule-specific protein in mycorrhizal roots whereas cleavage products of a GRAS TF predicted as a target of miR5204^*^ were also sequenced in mycorrhizal roots (Devers et al., 2011).

### Recent legume miRNAs linked to nodulation

Differential accumulation of miRNAs during rhizobial interactions and nodule development have been reported in several studies (Subramanian et al., 2008; Wang et al., 2009; Li et al., 2010). However, functional analyses of miRNAs remained rare and only two miRNAs, miR169, and miR166, were experimentally associated to nodule development before 2009 (Data Sheet 2; Combier et al., 2006; Boualem et al., 2008). During the last three years, a set of interesting studies have interconnected additional miRNAs to the regulatory networks that control nodulation (Data Sheet 2). D'Haeseleer et al. (2011) reported that over-expression of miR164, a conserved miRNA targeting *NAC1* TF in roots, affected nodule organogenesis in *M. truncatula*, presumably through deregulation of auxin responses. A functional analysis of several soybean miRNAs highly expressed in roots inoculated by symbiotic bacteria, first identified by Subramanian et al. (2008), was performed by Li et al. (2010): the conserved miR482 and five legume-specific miR1507, miR1511, miR1512, miR1515, and miR1521. Analysis of miRNA accumulation in the NOD49 nodulating mutants (mutated in the NOD factor receptor 1, NFR1) and the super-nodulation mutant NTS382 (impaired in NARK1, a receptor kinase involved in nodule auto-regulation pathway), pointed out that gma-miR1507, gma-miR1511, and gma-miR1512 expression was dependent on NFR1 or NARK1. Furthermore, miRNA over-expression in transgenic roots under the control of a constitutive or a nodulation-specific *ENOD40* promoters, showed that miR482, miR1512, and miR1515 positively regulated nodule number (Li et al., 2010). MiR1512 targets a Copine-like membrane protein that participates in cell signaling and transport. MiR482 and miR1515 repress disease resistance genes and/or *DCL2*, the DICER-like gene mainly related to defence against viruses. This could be related to the fact that symbioses share some common features with early defence responses (Ochman and Moran, 2001; Simon et al., 2009; Corradi and Bonfante, 2012; Marchetti et al., 2010; Bourcy et al., 2013; Peleg-Grossman et al., 2013). In *G. max*, 4 novel miRNAs (gma-new-miR4416a, gma-new-miR4416b, gma-new-mi13587, and gma-new-miR50841) were also reported as highly expressed in nodules (Turner et al., 2012). In addition, the accumulation of the corresponding targets in roots and nodules negatively correlated with the relative abundance of the miRNAs, suggesting that these genes may be specifically regulated in organs through miRNA spatial distribution (Turner et al., 2012). However, their functions remain unknown.

Very recently, the first identification of miRNAs from *Lotus japonicus* has been published by De Luis et al. (2012). These authors showed that miR397 is required for the establishment and maintenance of determinate nodules in *Lotus* but not for nodule organogenesis. The *snf* mutant (spontaneous nodulation mutant) inoculated with *Mesorhizobium loti* accumulated more miR397 than non-inoculated plants. One potential miR397 target in *M. truncatula* is homologous to the *A. thaliana* LACCASE10 (a copper-containing oxidase enzyme). In *A. thaliana*, miR397 was linked to nutrient interchanges between shoots and roots. During nodulation, nutrient exchanges occur between plant cells and bacteroids in nodules, and miR397 may be necessary to maintain a correct level of copper during this process (De Luis et al., 2012). Moreover, to maintain the nitrogen fixation rate, O_2_ concentrations must be regulated inside the nodule cells. Cu/Zn superoxide dismutase (SOD) scavenges superoxide radicals avoiding the inhibition of nitrogenase activity (Rubio et al., 2007). This enzyme depends on the availability of Cu^2+^ inside the cells and a decrease in Cu/ZnSOD expression level takes place when miR397 is over-expressed. This result is consistent with the fact that miR397 levels are higher in mature-senescent nodules than in younger ones, a stage where over-production of reactive oxygen species can be detected (Matamoros et al., 1999). Interestingly, miR397 expression is regulated by SPL7 in *Arabidopsis*. This TF also controls the expression of miR398, another conserved miRNA which negatively regulates Cu/ZnSOD genes (Mendoza-Soto et al., 2012). In addition, miR408 and miR857 were also regulated in response to Cu^2+^ through SPL7; although their level does not increase during nodulation (De Luis et al., 2012). In conclusion, miR397 and miR398 may participate to a complex regulatory mechanism that controls at least copper homeostasis in nodules. As indicated before, although miR397 has been reported in soybean and *L. japonicus* (Wang et al., 2009; De Luis et al., 2012) there is no evidence of this miRNA in *M. truncatula*. Thus, miR397 could be necessary for bacterial infection in determinate nodules like those from L. japonicas and *G. max* but not in the indeterminate nodules of *M. truncatula*. There are several differences between these nodule types including the persistence of the meristem and bacteroid differentiation and several genes were specifically associated to indeterminate nodules (Van de Velde et al., 2010). Differences in miRNAs between determinate and indeterminate nodules are consistent with the fact that both types of legumes do not respond equally to nodulation signals and show different morphology, physiology and responses to stress (Subramanian et al., 2007; Deinum et al., 2012; López-Gómez et al., 2012).

Finally, the role of miR171 in legumes, first suggested by Devers et al. (2011), emerged through three different reports. Lauressergues et al. (2012) and De Luis et al. (2012) showed that the miR171h and miR171c variants are fundamental to establish symbiotic mycorrhization and nodulation in *M. truncatula* and *L. japonicus*, respectively. When *M. truncatula* roots are infected by the AM fungus *Rhizophagus irregularis*, the increase in miR171h was followed by a concomitant decrease in the corresponding target *NSP2*. In addition, overexpression of miR171h led to a decrease in fungal colonization associated to the down-regulation of mycorrhizal marker genes (Lauressergues et al., 2012). In *L. japonicus*, De Luis et al. (2012) showed that, similarly to miR397, the miR171c isoform accumulated in inoculated *snf* mutants and that this miRNA variant was associated to nodule establishment and maintenance but not organogenesis. In both species, the specific miR171 isoforms studied were able to cleave the target transcript NSP2, a TF involved in NOD factor signaling. Furthermore, as *MtNSP2*, mt-miR171h was also early activated in response to cytokinins (Ariel et al., 2012). The emerging idea is that *NSP2* evolved in legumes to acquire specific functions during nodulation. Therefore, miR171h may be required for nodule but also for mycorrhiza establishment through *NSP2* regulation, a node involving cytokinin signaling. The common function of miR171 isoforms in nodulation and mycorrhization reinforces the idea that nodule development may have evolved from AM fungi interactions though a diversification and specialization process.

## Phased siRNA in legumes

Several siRNAs were identified showing “phasing” or processing cleavage of 21 nt derived from a long precursor. This is the case for the *TAS* genes that are targeted by different miRNAs as well as for the recently described phasiRNAs.

### TAS3 tasiRNA regulation is conserved in legumes

In *Arabidopsis thaliana*, 4 *TAS* genes have been well described (*TAS1* to *TAS4*). *TAS1* is target of miR173, while *TAS2* and *TAS4* are cleaved by miR828. Their miRNA-dependent cleavage requires the action of AGO1 (Peragine et al., 2004). In contrast, *TAS3*-tasiRNA biogenesis depends on miR390 and AGO7 (Montgomery et al., 2008). The cleaved *TAS* transcripts are used as templates by RDR6 in complex with the Suppressor of Gene Silencing III protein (SGS3). The resulting long dsRNA is cleaved into secondary 21 nt siRNAs respecting a certain phase due to the processivity of DCL4 (Vaucheret, 2005). Hence, DCL4 generates several phased tasiRNA that will be loaded into an AGO1-containing RISC complex to target complementary mRNAs. Interestingly, except for miR390, the miRNAs that participate in the biogenesis of tasiRNA are 22 nt in length (Cuperus et al., 2011). This 1 nt difference of the loaded miRNA has been proposed to be the clue to direct AGO1 from mRNA cleavage without any amplification (like all 21 nt miRNAs) to the production of secondary siRNAs. Recently, Manavella et al. (2012) proposed that the asymmetry provoked by the loading of a 22 nt miRNA would alter AGO1 conformation and, in this way, it might condition the capacity to trigger transitivity via RDR enzymes.

The most conserved *TAS* gene in plants is *TAS3* which is targeted by miR390, a 21 nt miRNA. *TAS3* RNA presents two miR390 binding sites, however only the 3′ site is cleaved following base pairing with miR390 (Montgomery et al., 2008). Furthermore, miR390 is loaded on a specific AGO7-containing complex to trigger tasiRNA production on cleaved *TAS3* transcripts. *TAS3*-derived tasiRNAs target specific members of the ARF family: ARF2, ARF3 and ARF4. These TFs are involved in auxin responses and therefore in many developmental stages (Jouannet et al., 2012), notably lateral root formation a process which may be linked to symbiotic interactions in legume roots (Marin et al., 2010). In *M. truncatula*, Jagadeeswaran et al. (2009) reported that, among the four *Arabidopsis TAS* genes, only *TAS3* and its *ARF* targets are conserved. In addition, mutants affected in AGO7 and SGS3, two main enzymes involved in *TAS3*-tasiRNA biogenesis were found in *L. japonicus*. These mutants, called *Ljrel* (REduced Leaflet), display similar leaf phenotypes, with abaxialized leaflets and lower leaflet numbers as well as defects in flower development (Peragine et al., 2004; Yan et al., 2010). *Ljrel1* mutants (affected in SGS3 function) down-accumulate *TAS3*-tasiRNAs leading to the misregulation of their ARF targets (Yan et al., 2010). However, although *TAS3*-siRNA are involved in the quantitative regulation of root lateral organogenesis in *A. thaliana* (Marin et al., 2010), nodulation phenotypes were not yet reported in these mutants.

### The phased short interfering RNA (phasiRNA): a new important class of siRNA in legumes

As mentioned before, 22 nt miRNAs (instead of their 21 nt corresponding variants) may affect the conformation of AGO1 and trigger the production of secondary phased siRNA of 21 nt (Chen et al., 2010; Cuperus et al., 2011). In *Arabidopsis*, similar abundances of the mature 21 nt and 22 nt variants were found for several miRNA families, like miR173, miR828, miR472 (Cuperus et al., 2011) or miR319 and miR771 (Chen et al., 2010). The 22 nt variants are loaded into an AGO1-containing RISC complex and triggers cleavage of their complementary targets. However, like for *TAS* transcripts, cleavage products are transformed into long dsRNA by the RDR6/SGS3 complex and then spliced into phased secondary siRNAs, mainly through DCL4 (Cuperus et al., 2011). Such a mechanism has also been shown in tobacco (Li et al., 2012). In rice, biogenesis of phased siRNAs occurred through a similar mechanism, which involves 24 nt siRNAs and a DCL3 isoform instead of DCL4 (Song et al., 2012). Indeed in the *osdcl4* mutant, 24 nt phased smRNA still accumulate thanks the action of the OsDCL3b protein. In that species, two 22 nt miRNAs (miR2118 and miR2275) were necessary to induce the secondary production of 24 nt phased siRNAs. Hence, at least in rice, the production of phased siRNAs depends on a particular DCL isoform, different from DCL4 and issued from DCL3 duplication and specialization (Song et al., 2012). Those results indicate that 22 nt miRNAs may have been selected to generate both 21 or 24 phased siRNA in plants during evolution.

Last year, Zhai et al. (2011) studied in detail the diversity of 21 nt phased siRNAs in several smRNA libraries from soybean and *M. truncatula*. These siRNAs, called phasiRNA, derive from *PHAS* genes, that are primarily cleaved by 22 nt mature miRNAs. These authors identified 114 and 41 *PHAS* loci in *M. truncatula* and soybean, respectively. In *M. truncatula*, 112 *PHAS* loci corresponded to protein-coding genes and 2 to intergenic regions, while 26 and 15 were identified as protein-coding genes or intergenic respectively in soybean. Around 68% of the *PHAS* loci in *M. truncatula* contained one 22 nt miRNA binding site, and most of them were triggered by one of the four 22 nt miRNAs, miR1501, miR1509, miR2109, and miR2118, which are predominantly abundant in this species. Among *PHAS* loci which contained two 22 nt miRNA binding sites, those authors found an *APETALA2* (*AP2*)-like gene, which possess one cleavage site for miR172 and a predicted non-cleavable miR156 target site, resembling the two miR390 complementary motives in TAS3. In soybean and *L. japonicus*, however, *AP2* orthologous only present one miR172 binding site. Thus, the acquisition of the second (miR156) miRNA binding site in *MtAP2* certainly happened recently in evolution (Zhai et al., 2011) and this may have consequences on the generation of secondary siRNAs.

Interestingly, some genes coding for enzymes involved in smRNA pathways have been identified as *PHAS* loci (Zhai et al., 2011). In soybean, *GmSGS3a* transcripts are targets of miR2118. *DCL2* mRNAs are cleaved by miR1507 or miR1515 in *M. truncatula* and soybean, respectively. This suggests that in those legumes, *DCL2* genes evolved independently to acquire similar regulation by a 22 nt miRNA and production of secondary phasiRNAs. The targeting activity of the phasiRNAs on genes involved in smRNA biogenesis may also suggest a feedback mechanism, reminiscent of the regulation of *AGO1* and *DCL1* by miR168 and miR162, respectively. Hence, 22 nt miRNAs and phasiRNAs generated by *DCL2* or *SGS3* may control these targets which are essential for their own production. Jagadeeswaran et al., 2009 identified TIR-NBS-LRR proteins as mt-miR2118 targets. In addition, recently, it has been shown that most *PHAS* loci targeted by miR1507 and miR2118 encode NBS-LRR resistance proteins (Zhai et al., 2011) leading the authors to suggest an important role of these miRNAs in the control of biotic interactions in legumes, including symbiotic nodulation. However, these miRNAs are not specific from legumes and similar regulations of NBS-LRR genes by 22 nt miRNAs were described in non-legumes, like *Nicotiana benthamiana* and tomato (Li et al., 2012). In the first species, ten 22 nt miRNA families target a large set of resistance genes. Among them, Nta-miR6019, also found in other solanaceaous species, conferred resistance to the TMV (Tobacco Mosaic Virus). In tomato, the 22 nt variant of miR482 cleaves six NBS-LRR proteins, and at least two of them contain a second miR482 binding site (Shivaprasad et al., 2012). Infection of tomato plants by Turnip crinkle virus (TCV), Cucumber mosaic virus (CMV) or Tobacco rattle virus (TRV) increased the expression of NBS-LRR by decreasing miR482 accumulation (Shivaprasad et al., 2012). The authors proposed that, when the pathogen is absent, phasiRNAs block the NBS-LRR expression in order to reduce energetic cost to the plant. These data suggested that the control of disease resistance gene expression by 22 nt miRNAs through secondary production of phasiRNAs evolved early in plants and is not exclusive from legumes. As in nature, bacteria and virus pathogens have co-evolved with plants and gene-for-gene relationship exist (Jones and Dangl, 2006), virus infection could trigger a suppression of NBS-LRR-targeting miRNA biogenesis which leads to increase NBS-LRR expression and defense reactions. Symbiotic interactions share some common features with pathogenesis (Soto et al., 2009) and co-evolution may have favored the maintenance of miRNA targeting of NBS-LRR phased loci in symbiotic interactions.

In addition to the function of new miRNAs evolved in legumes and regulated in symbiotic interactions, functional analyses of those 22 nt miRNAs and their derived phasiRNAs in nodulation and pathogen responses remain an interesting challenge for the next years. Furthermore, the 24 nt heterochromatic siRNAs generally associated to the establishment of epigenetic patterns in different processes have been largely remain a forgotten aspect of smRNAome diversity in legumes.

## Small RNA pathways in legumes

Despite the diversity of smRNA in different plants, the core of their biogenesis pathways depends generally on DCL and AGO proteins (Parent et al., 2012). DCLs and AGOs are encoded by multigenic families of 10 and 4 members in *A. thaliana* and 19 and 6 members in rice (Kapoor et al., 2008). In comparison, vertebrates, nematodes and yeast contain only one DCL, whereas insects, protozoa and fungi have two DCL isoforms. Plants thus globally show an important gene diversification of DCLs and AGOs, that led to the specialization of certain isoforms in different smRNA pathways (Hutvagner and Simard, 2008; Murphy et al., 2008; Liu et al., 2009). Although previous works have been published about phylogenetic and evolutionary aspects of these enzymes in plants, to our knowledge, there is no published global analysis of DCL and AGO proteins focused on model legumes. Even though their genome sequences are not yet completely finished, a very large portion is available in genomic and EST databases for database mining. For this, we carried out TBLASTX analyses to identify the orthologs of *Arabidopsis* DCL and AGO proteins in the three legumes for which large genomic databases are available: *Lotus japonicus* (Miyakogusa.jp 2.5, Sato et al., 2008), *Medicago truncatula* (Mt 3.5.1, Young et al., 2011), and *Glycine max* (Glyma1.181, http://www.plantgdb.org/GmGDB/). In addition, we compared our results to those already published for rice (Kapoor et al., 2008) and poplar (*Populus trichocarpa*, Margis et al., 2006). The complete list of DCL and AGO genes is given in Data Sheet 3.

### DICER-like proteins in legumes

In *A. thaliana*, the four DICER-like proteins produce differently sized smRNAs and have complex relationships (Gasciolli et al., 2005). DCL1 is mainly involved in miRNA biogenesis. It processes the miR/miR^*^ duplexes from imperfect fold back stem-loops of the pri-miRNA precursors, while DCL2, DCL3 and DCL4 are more generally responsible for generating siRNA from dsRNA originating from exogenous elements, natural antisense genes, TAS (and likely PHAS) transcripts or repeated heterochromatic regions. *dcl2/3/4* triple mutants showed a reduction in siRNA production, but there was no change in miRNA populations, confirming that DCL1 is the main enzyme involved in miRNA biogenesis. In addition, in rice and *A. thaliana*, DCL1 loss of function causes embryo lethality (Liu et al., 2005; Song et al., 2011). However, in *A. thaliana*, smRNAs derived from inverted repeats that fold into imperfect hairpins were also more abundant in *dcl2/3/4* triple mutant (Henderson et al., 2006). This observation correlates with the fact that DCL1 is necessary for the accumulation of the Cauliflower mosaic virus-derived siRNA that also derive from an imperfect fold back RNA structure (Dunoyer et al., 2007). Thus, DCL1 is specialized in the production of miRNA or siRNA of 21 nt from imperfect fold backs structures (Chapman and Carrington, 2007). In *A. thaliana*, Gasciolli et al. (2005) showed that the accumulation of tasiRNA targets increased in *dcl4* and *dcl3/dcl4* mutants, while *dcl2/dcl3* and *dcl3/dcl4* present stochastic developmental phenotypes due to the lack of accumulation of heterochromatic siRNA-directed marks. On the other hand, analysis of *dcl* double and triple mutants pointed out that DCL2, DCL3 and DCL4 have compensatory functions among them (Gasciolli et al., 2005; Henderson et al., 2006). For instance, in *dcl3* mutants, DCL2 and DCL4 are able to produce 22–21 nt siRNAs from DCL3 substrates (Gasciolli et al., 2005). In addition, in viral siRNA biogenesis, DCL4 acts in a hierarchical manner with DCL2 (Rajagopalan et al., 2006), using the DCL2-dependent 22 nt siRNA to trigger secondary siRNA biogenesis (Chen et al., 2010).

Other plants than *A. thaliana* generally possess more than 4 DCLs. For instance poplar (*P. trichocarpa*) has 5 DCLs (PtDCL1, PtDCL2a, PtDCL2b, PtDCL3, and PtDCL4, Margis et al., 2006) and rice contains 6 DCLs (OsDCL1, OsDCL2a, OsDCL2b, OsDCL3a, and OsDCL3b and OsDCL4) (Kapoor et al., 2008). Although Kapoor et al. (2008) reported 3 different *OsDCL1*-like genes, we assume that the so-called *OsDCL1b* and *OsDCL1c* genes in fact encode another kind of ribonuclease III, closed to DCLs, the RNase Three Like (RTL) proteins. Thus, only the *OsDCL1a* gene was taken into account in our study. As previously reported, in rice, duplication of *DCL3* has been followed by a specialization of the two isoforms. Indeed, OsDCL3a classically triggers hc-siRNA biogenesis while OsDCL3b, which shows a specific expression in early stages of seed development (Kapoor et al., 2008), is mainly involved in the biogenesis of 24 nt phased siRNA (Song et al., 2012). Accordingly to this, inactivation of *OsDCL3b* by RNA interference affected the accumulation of 24 nt phased siRNAs but not of 24 nt hc-siRNAs (Song et al., 2012).

**Figure 3:**
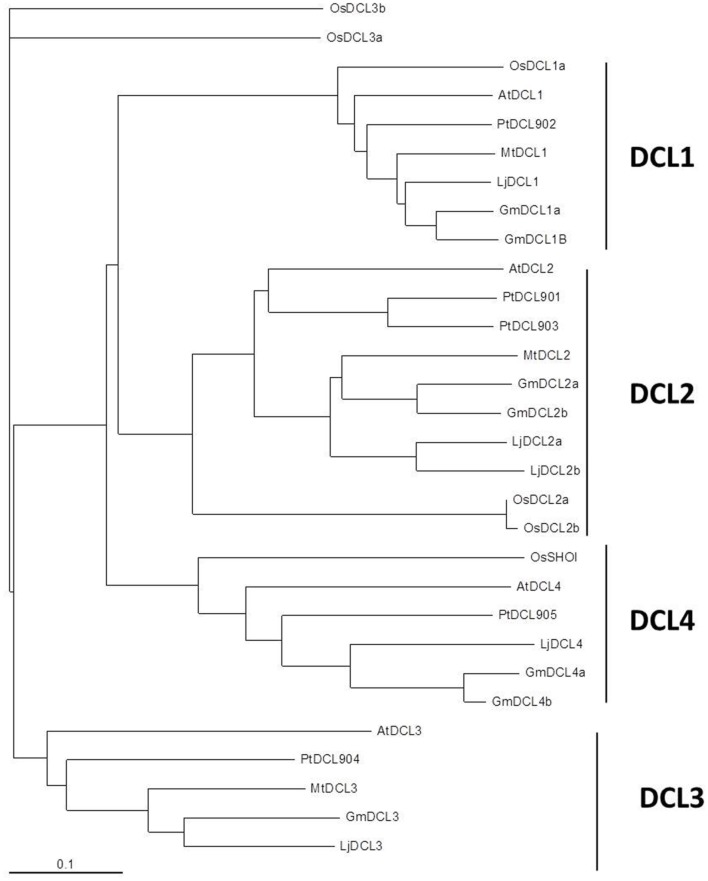
**Phylogenetic tree of DICER-like proteins in *M. truncatula, L. japonicus, G. max, A. thaliana, P. trichocarpa*, and *O. sativa***. Full length sequences of the predicted DCL proteins from *A. thaliana, P. trichocarpa*, and *O. sativa* were retrieved from Genbank (NCBI). DCL from *M. truncatula*, soybean (*G. max*) and *L. japonicus* were searched by tBLASTX in genomic sequence databases: legumes *Lotus japonicus* (Miyakogusa.jp 2.5, Sato et al., 2008), *Medicago truncatula* (Mt 3.5.1, Young et al., 2011), and *Glycine max* (Glyma1.181, http://www.plantgdb.org/GmGDB/), and named according to their similarity with *A. thaliana* proteins or according to previous publications (correspondences given in Data Sheet 3). Phylogenetic trees were constructed thanks to T-Coffee software. The phylogenetic tree was generated with MEGA4.0 software using the Neighbor joining tree (Capitão et al., 2011). The four DCL clades are indicated in the right part of the figure.

In legumes, Curtin et al. (2012) reported 6 *DCL* genes in *G. max* (*GmDCL1a, GmDCL1b, GmDCL2a, GmDCL2b, GmDCL4a*, and *GmDCL4b*). In *M. truncatula*, both Capitão et al. (2011) and Young et al. (2011) identified one homolog for *DCL1, DCL2* and *DCL3* genes, but pointed out the absence of *DCL4* homolog. By searching in *L. japonicas* genomic sequences, we identified five *LjDCL* genes: *LjDCL1, LjDCL2a* and *LjDCL2b, LjDCL3* and *LjDCL4* (Data Sheet 3). To construct a phylogenetic tree of DCLs (Figure 3), only the complete predicted proteins were retained. According to our analysis, DCL proteins were clearly grouped into four monophyletic groups, each corresponding to one of the four AtDCLs, and with high sequence conservation between species. Like in non-legumes, only one *DCL1* gene was found in *M. truncatula* and *L. japonicus*, while two *GmDCL1* genes are present in soybean. The functional significance (if any) of this specific event of *DCL1* duplication in soybean remains to be elucidated. In contrast, like in many angiosperms, a duplication of *DCL2* was observed both in *L. japonicus* and *G. max*. According to Margis et al. (2006), this duplication event took place before the divergence between *P. trichocarpa* and *A. thaliana*. However, *M. truncatula* apparently contains a unique *DCL2* gene. As the genome sequence is not fully complete in that species, the most probable explanation may be that the second *DCL2* locus has not yet been sequenced. However, we were also not able to identify expressed sequences corresponding to a putative second *DCL2* homolog, although the *M. truncatula* EST database is very large (TIGR MtGi11.0). In contrast to rice and maize, that shared a DCL3 duplication (Margis et al., 2006; Kapoor et al., 2008), only one *DCL3*-like gene was described in the three model legumes, even in the *G. max* paleopolyploid genome (Gill et al., 2009). Curtin et al. (2012) reported a second DCL3 but the presence of an in-frame stop codon and the absence of several DCL characteristic domains led to consider it as a pseudogene. These data thus reinforces the idea that *DCL3* diversification may be specific to monocots.

To our opinion, the absence of *DCL4* sequences in the *M. truncatula* genome annotation (Young et al., 2011) is certainly due to its still incompleteness. Indeed, we were able to found a genomic contig (contig_162690_1.1) which contains a putative incomplete *DCL4* gene. Unexpectedly, two DCL4 homologs were found in soybean, that shared a similarity of 79.9% and 80.3% at nt and amino acid levels, respectively. The genome of soybean has suffered two rounds of large scale-sequence or segmental duplication (Gill et al., 2009), thus the existence of two *DCL1* and two *DCL4* genes in soybean may derive from a segmental duplication event. This idea is reinforced by genome browser data, showing that both *DCL4*s are flanked by genes coding homologous proteins (at 5′, Glyma13g22420 and Glyma17g11220, which are annotated as MCM5 minichromosome maintenance family protein; and at 3′, Glyma13g22460 and Glyma17g11250, annotated as Phosphoinositide binding protein).

**Figure 4:**
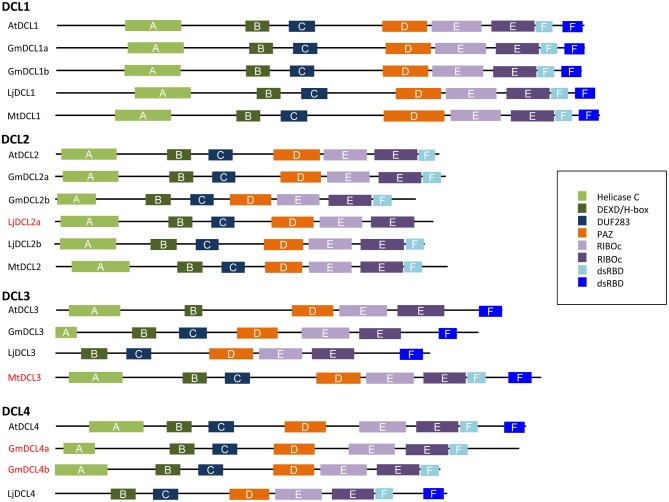
**Conserved domains in DCL proteins in *M. truncatula, L. japonicus, G.‘max* and, *A. thaliana***. Conserved domains searched using Simple Modular Architecture Research Tool-SMART version 7 (Letunic et al., 2012). Helicase-C, DEAD-helicase box (DEXD/H-box), DUF283, RNaseIII (RIBOc), double-strand RNA-binding domains (dsRBD), and Piwi/Argonaute/Zwille (PAZ).

To further investigate the diversity of legume DCLs, we compared their conserved domains using Simple Modular Architecture Research Tool-SMART version 7 (Letunic et al., 2012; Figure 4). In plants, DCLs contain six types of conserved domains: helicase-C, DEAD-helicase box (DEXD/H-box), DUF283, RNase III (RIBOc), double-strand RNA-binding (dsRBD) and PAZ (Piwi/Argonaute/Zwille) domains (Margis et al., 2006; Murphy et al., 2008). Two RNase III domains are required to constitute an intramolecular dimer, necessary for the cleavage of dsRNA substrates. According the DCL, one or two dsRBD domains are present. The PAZ domain participates in protein-protein interactions and, because of its ubiquitous presence, a role in the interaction between DCLs and other proteins was suggested, thus guiding template recognition (Carmell and Hannon, 2004). The DEAD-helicase box domain seems to be essential in DICER auto-regulation. In humans, removal of this domain increases the cleavage rate of DICER proteins. DUF283 domain may be involved in the selection of the different smRNA biogenesis pathways as it recognizes the asymmetry of dsRNA substrates (Liu et al., 2009).

In the plants studied so far, DCL1 and DCL4 proteins possess all functional domains, including two dsRBD domains. As expected, in legumes, DCL1s were also highly conserved and shared all domains necessary for miRNA biogenesis (Gasciolli et al., 2005; Parent et al., 2012). Unexpectedly, only one dsRBD domain was found in the two *GmDCL4* isoforms. However, a *DCL4*-like gene coding for a protein containing one dsRDB domain is located 2654 bases from the presumed stop codon of *GmDCL4a*, suggesting a problem of annotation. In contrast to DCL1 and DCL4, DCL2 and DCL3 isoforms are much more variable, in particular in their number of dsRDB domains (Cerutti and Casas-Mollano, 2006; Margis et al., 2006). In rice, for instance, both DCL2 isoforms, although functional, contain only one dsRBD domain (Margis et al., 2006). In addition, OsDCL3a has two classic dsRBD domains, whilst OsDCL3b lacks one. Although Margis et al. (2006) reported that AtDCL3, PtDCL3, and OsDCL3b possess two dsRDB domains, according to our analyses using the SMART version 7 software (http://smart.embl-heidelberg.de/), those proteins appear to contain only one typical dsRDB domain. In legumes, we also noticed such variability with two dsRDB domains in MtDCL3 and only one in GmDCL3 and LjDCL3. In our opinion, the more striking observation in term of domain composition was the lack of any canonical dsRBD in LjDCL2a. Although a problem of annotation remains an hypothesis, it is possible that the DCL2 function is mainly assumed by LjDCL2b in *L. japonicus*.

Presence/absence as well as number or type of dsRBD domains have been proposed to be important criteria to determinate the substrate specificity and the interaction of AGOs with associated proteins, including DCLs, of the smRNA pathways (Hiraguri et al., 2005; Eamens et al., 2009). Fusion GFP proteins of DCL1, DCL4, and other proteins involved in smRNA pathways like HYPONASTIC LEAVES (HYL1) and DRB (Double-strand RNA Binding) proteins suggested co-localization in specialized ribonucleoproteins in *A. thaliana* (Hiraguri et al., 2005). It has been proposed that dsRBD and PAZ play roles in recognizing and processing the RNA substrates while dsRBD play additional functions by interacting with other proteins of smRNA biogenesis pathways (Hiraguri et al., 2005; Eamens et al., 2009). It will be interesting to investigate the precise function of the *DCL* genes that have been duplicated in some legumes or present different dsRBD compositions in relation to smRNA biogenesis.

### Argonautes proteins and their conserved domains in legumes: another complex story

AGOs are RNA binding proteins that are key effectors of the RISC complexes. AGO-like proteins have been found in bacteria, archea and eukaryotes (Hutvagner and Simard, 2008). *A. thaliana*, rice and maize possess 10, 19, and 18 AGO proteins respectively (Kapoor et al., 2008; Vaucheret, 2008; Qian et al., 2011). Similarly to DCLs, certain AGOs show functional redundancy (Vazquez et al., 2010). Plant AGO proteins fall into four distinct clades with different properties in term of smRNA recognition based on sequence similarity: AGO1/10, AGO5, AGO2/3/7, and AGO4/6/8/9 (Mallory and Vaucheret, 2010; Czech and Hannon, 2011). AGO proteins show preferences for the nucleotide placed at the 5′ end of the smRNAs as shown by immunoprecipitation of AGO complexes in *Arabidopsis* (Mi et al., 2008; Montgomery et al., 2008; Takeda et al., 2008; Havecker et al., 2010). For instance, AGO1 and AGO10 bind 21 nt (and eventually 22 nt) miRNAs with a 5′ uridine; AGO4/6/9 and AGO2/3 prefer 5′ adenine residues on 24 and 21 nt siRNA respectively, while AGO5 binds smRNAs with a 5′ cytosine (Manavella et al., 2011). However, exceptions to this nucleotide preference may exist and these rules are not absolute. In addition, a specific AGO, AGO7, *Arabidopsis* essentially binds the conserved miR390, which contains a 5′ adenine in *Arabidopsis*. Changing this residue into a cytosine did not produce a difference in AGO7 preference for miR390 (Montgomery et al., 2008) showing that the 5′ end is not the only determinant of AGO binding, at least in this case.

**Figure 5:**
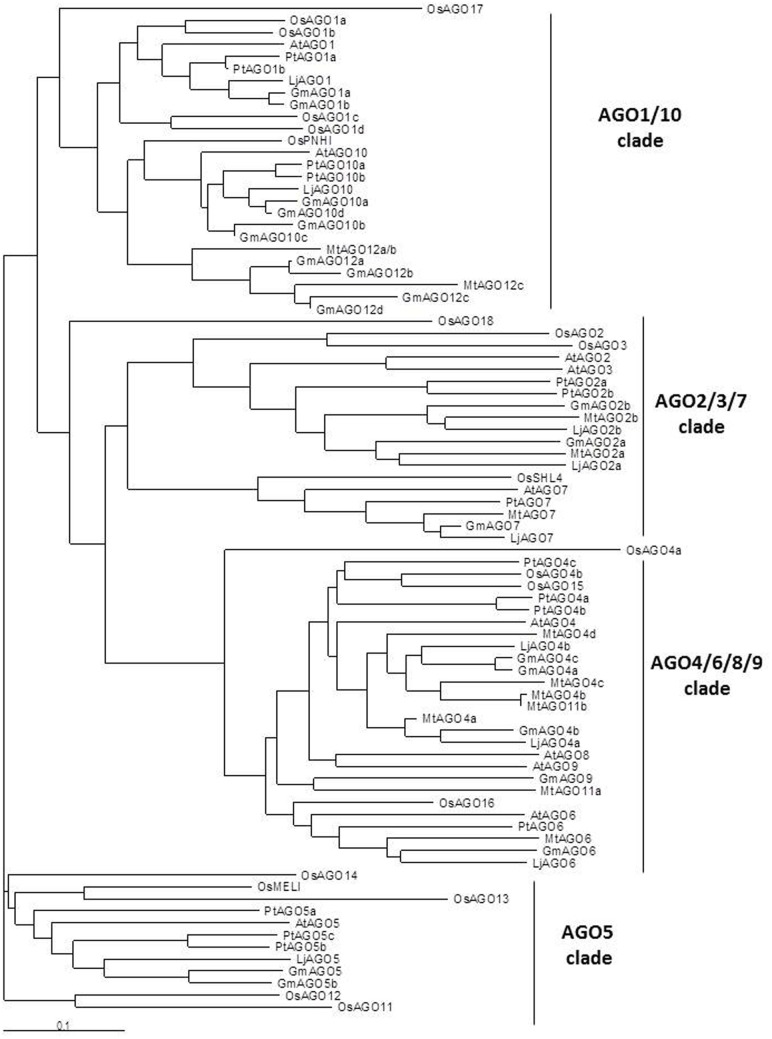
**Phylogenetic tree of AGO proteins in *M. truncatula, L. japonicus, G. max, A. thaliana, P. trichocarpa*, and *O. sativa***. Full length sequences of the predicted AGO proteins from *A. thaliana, P. trichocarpa* and *O. sativa* were retrieved from Genbank (NCBI). DCL from *M. truncatula*, soybean (*G. max*) and *L. japonicus* were searched by tBLASTX in genomic sequence databases: legumes *Lotus japonicus* (Miyakogusa.jp 2.5, Sato et al., 2008), *Medicago truncatula* (Mt 3.5.1, Young et al., 2011), and *Glycine max* (Glyma1.181, http://www.plantgdb.org/GmGDB/), and named according to their similarity with *A. thaliana* proteins or according to previous publications (correspondances given in Data Sheet 3). Phylogenetic trees were constructed thanks to T-Coffee software. The phylogenetic tree was generated with MEGA4.0 software using the Neighbor joining tree (Capitão et al., 2011). The four AGO clades are indicated in the right part of the figure.

Our search by TBLASTX pointed out the presence of 21, 12, and 9 putative AGOs in *G. max, M. truncatula* and *L. japonicus*, respectively, as well as 14 homologs in *P. trichocarpa* (Tuskan et al., 2006; *Populus trichocarpa* v3.0, DOE-JGI, http:://www.phytozome.net/poplar). A previous search in *M. truncatula* (Capitão et al., 2011) already described 12 AGOs based on the MtV3.0 genome (correspondence between ours and theirs AGOS is given in Data Sheet 3). As shown in the AGO phylogenetic tree (Figure 5), legumes AGOs clearly fall into the four clades described in *Arabidopsis*. In soybean, homologs for each AtAGO were found, except AGO8. In fact, the eventual absence of AGO8 is shared by the three legumes, despite that the genomes are not complete. This may be consistent with Takeda et al. (2008) who suggested that *AGO8* may be a pseudogene in *A. thaliana*. Several AtAGO homologs could not be identified in present *M. truncatula* and *L. japonicus* genomic databases. For instance, AGO3 and AGO9 are absent both in *Lotus* and *M. truncatula* genomic databases (Capitão et al., 2011; Young et al., 2011). However, according to its strong similarity with the soybean AGO9, we propose to rename MtAGO11a (Capitão et al., 2011) in MtAGO9. In addition, as AGO2 and AGO3 belong to the same clade, it is possible that the absence of AGO3 in the two model legumes is compensated by the presence of an additional AGO2 homolog (two AGO2 homogs instead of AGO2 and AGO3). In *Arabidopsis*, several evidences from *Atago2* mutants pointed out that AGO2 but not AGO3 plays fundamental roles in defense against particular virus infections (Harvey et al., 2011; Wang et al., 2011), suggesting that they may have specialized functions. On the other hand, expression analysis suggest that AGO2 and AGO3 are more expressed in seeds than other tissues and accumulation of smRNA species is similar in *ago2* and *ago3* mutants (Takeda et al., 2008). These latter experiments support that there is functional redundancy of AGO2 and AGO3 in *Arabidopsis*, notably in seeds.

Because of their fundamental roles in miRNA function, absence of clear homologs of AGO1 and AGO10 genes in *M. truncatula* databases was much more striking. In *Arabidopsis*, functional analyses suggested that AGO1 mainly acts through slicing and controls miRNA function. On the other hand, AGO10 acts through translational repression of the miRNA targets and belongs to the same clade (for references see Manavella et al., 2011). As *Atago10* mutants are affected neither in gene silencing nor in smRNA accumulation (Takeda et al., 2008), and *ago1* single mutant and *ago1 ago10* double mutants are embryo lethal (Lynn et al., 1999) it seems that they are not functionally redundant. However, partial functional redundancy between AGO1 and AGO10 proteins has been described in *Arabidopsis* (Maunoury and Vaucheret, 2011). AGO10 apparently preferentially associates with members of the miR165/166 family and some miRNA/miRNA^ ^ structural features seemed to be essential for its activity. AGO10 protein might thus act avoiding miR165/166 loading into AGO1, and hence attenuating the action of these miRNAs by cleavage (Ji et al., 2011; Manavella et al., 2011). In *M. truncatula*, Capitão et al. (2011) first identified an expressed sequence contig (TC126810, TIGR MtGi9.0) encoding a putative AGO1 homolog. However, in the last version of TIGR (MtGi11.0, March 2011), this TC was split into two contigs (TC188472 and TC194233), which in fact correspond to the so-called MtAGO12a/b genes. Although these AGO12 isoforms fall into the AGO1/AGO10 clade, they are more closely related to AGO10 (Figure 5). Thus, the absence of AGO1, but also of AGO5, in *M. truncatula* databases remains intriguing and may be due to the lack of completion of the *M. truncatula* genome.

Vaucheret (2008) and Kapoor et al. (2008) reported that new AGO genes arise from gene duplication events. This type of gene diversification clearly appears in the phylogenetic tree which shows that, for many AtAGOs, more than one homolog was found in nearly all plant species we compared. Looking at the phylogenetic relationships inside the AGO4/6/8/9 clade, AGO4 diversification was important in all species with 2–4 homologs. In addition, AGO4s from the three model legumes appeared clearly separated from the corresponding homologs in non-legumes. A divergence in smRNA biogenesis proteins has been observed in vertebrates, invertebrates and plants suggesting a lineage-specific modification of gene regulation by smRNAs, and pointing out to the plasticity of genome for the evolution of novel regulatory networks (Murphy et al., 2008). In this case, the possibility of an AGO4 evolution in legumes toward an adaptation of regulatory smRNA network and symbiotic interactions may be worth considering.

To further analyse putative functions of AGOs, we compared their conserved domains: MID, PIWI, PAZ, and DUF1787 domain of unknown function (Vaucheret, 2008). These domains are linked to different activities of AGOs: the MID domain binds the smRNA 5′ end (Wang et al., 2009; Parker, 2010); The PIWI domain is responsible of the catalytic activity of AGO proteins (Baumberger and Baulcombe, 2005); the PAZ domain is necessary to recognize the 3′ end of the smRNA for loading into the RISC and determines the AGO specificity. Among the identified domains in legume AGOs (Data Sheet 4), only some appeared unusual. For instance, GmAGO10c and MtAGO4a lacked PAZ and DUF1785 domains, suggesting that they are pseudogenes. On the other hand, MtAGO12c (Medtr2g059590, Capitão et al., 2011) had no PIWI domain. For that, we assume that this protein, even if it also belongs to the AGO1/AGO10 clade, may certainly not play similar functions than AGO1. Again, more precise analyses of the corresponding genomic regions and completion of the genome sequencing are required to support the absence of these sequences as errors in gene annotation due to the presence of very long introns for instance may explain some of the difficulties to detect gene homologs in legumes.

Finally, we decided to compare more precisely the sequence of the PIWI domains in legume AGOs (Data Sheet 5). Indeed, activity of the PIWI domain has been associated to the presence of conserved catalytic residues, in particular three metal-chelating amino acid residues Asp-Asp-His (DDH). Lack of one of these residues has been reported to alter AGO slicing but not its binding activity (Qi et al., 2006; Kapoor et al., 2008; Nowotny and Yang, 2009). For instance, AGO4 proteins with a modified DDH motif still bind siRNAs but lose their endonuclease activity (Qi et al., 2006). However, in *Arabidopsis*, the His residue of AGO2 and AGO3 may be replaced by Asp (DDD) without affecting their endonuclease activity (Baumberger and Baulcombe, 2005; Montgomery et al., 2008) and the same happened for 9 AGO proteins in rice (Kapoor et al., 2008). As shown in Data Sheet 1, all legume AGOs with a PIWI domain present either a DDH or a DDD motif, like in *Arabidopsis*. In addition, Baumberger and Baulcombe (2005) reported that a conserved Histidine residue at position +800 in the PIWI domain of AGO1 was required for its endonuclease activity, at least *in-vitro*. As expected, according to their role in RNA-dependent DNA methylation rather than RNA cleavage, AtAGO4/6/8/9 proteins contain another amino-acid residue, an alanine (A), a serine (S) or a proline (P), at this conserved position. We thus looked for the presence of the conserved H_800_ residue in legume AGOs. Like in *A. thaliana*, all AGO4, AGO6 and AGO9 proteins had no H_800_. Unexpectedly, the same is true in MtAGO12a/b proteins, again suggesting that they may not replace AGO1 cleavage function in *M. truncatula*, but may rather play similar translational regulatory functions to AGO10 (Zhu et al., 2011). Interestingly, AGO2b proteins of the three model legumes present an asparagine residue (N) instead of the H. As this amino-acid change did not occur in *Arabidopsis*, we wondered whether this substitution was found in other non-legumes species. Blasting the PIWI domain of AtAGO2 with the corresponding domains of AGO2 in rice and poplar, no changes in H_800_ was observed. In addition, when we analyzed all AGOs in both species, the H_800_ residue was never replaced by a N, although other amino-acids may be found such as Cysteine (C) in OsAGO17 or Glutamine (Q) in PtAGO4c. The presence of this N residue at a conserved key position of AGO2b proteins thus suggests a specific but conserved role of this isoform in legumes.

## Concluding remarks

Since five years, the rapid progress of genomic technologies has allowed the construction and high-throughput sequencing of a very large set of smRNA libraries in model legumes, in particular from *M. truncatula* and *G. max*. Compilation of data from miRBase and smRNA deep sequencings revealed the largest set of novel miRNA families ever observed in a plant family. Few conserved and legume-specific miRNAs have been functionally studied. Further analysis of the legume miRNAs as well as phased siRNAs and their putative targets will be essential to better understand the role of smRNAs in the control of key processes during nodule development, nitrogen fixation and defense against pathogens.

On the other hand, DCL and AGO proteins are key players in smRNA biogenesis and functions, which are highly conserved processes in plants. In legumes, many DCL and AGO proteins, like DCL2, AGO2, AGO4, AGO10 present a huge diversification, in comparison to *Arabidopsis thaliana*. However, in some legume models, highly conserved plant DCL and AGO formshave not been identified until now (e.g., MtDCL4 or MtAGO1/AGO10) or present a non-conventional domain composition. Further exhaustive genome analysis may be necessary to confirm the absence of those genes. Further analysis of DCL and AGO isoforms in legumes are necessary to clarify whether duplication of some isoforms could be correlated with a specialization in function and/or expression in legumes, in particular during symbiotic or pathogenic interactions.

### Conflict of interest statement

The authors declare that the research was conducted in the absence of any commercial or financial relationships that could be construed as a potential conflict of interest.
