# Supplementary material for: Small RNA pathways and diversity in model legumes: lessons from genomics
Source: Front Plant Sci. 2013 Jul 10;4:236. doi: 10.3389/fpls.2013.00236 (PMC3707012; doi:10.3389/fpls.2013.00236)
Supplement: Supplementary file 1 [file DataSheet1.PDF]

Publication	Species	Number of libraries	Technology
Sunkar et al., 2008	682 species (including Gma, Mtr, Lja)		*in silico*
Szittya et al., 2008	Mtr	2	Solexa
Jagadeeswaran et al., 2009	Mtr	1	454
Lelandais-Brière et al., 2009	Mtr	2	454
Devers et al., 2011	Mtr	2	Solexa
Wang et al., 2011	Mtr	2	Solexa
Zhai et al., 2011	Mtr	8	Solexa
Zhou et al., 2012	Mtr	2	Solexa
Chen et al., 2012a	Mtr	3	Solexa
Chen et al., 2012b	Mtr	2	solexa
Subramanian et al., 2008	Gma	2	454
Zhang et al., 2008	Gma		*in silico*
Wang et al., 2009	Gma	1	manual
Joshi et al., 2010	Gma	4	solexa
Song et al., 2011	Gma	1	solexa
Wong et al., 2011	Gma	2	454
Kuchelski et al., 2011	Gma	8	Illumina
Radwan et al., 2011	Gma	4	SOLID
Li et al., 2011	Gma	4	Illumina
Zhai et al, 2011	Gma	7	Illumina
Turner et al., 2012	Gma	idem Subramanian et al., 2008	idem Subramanian et al., 2008
De Luis et al., 2012	Lja	3	454

Data Sheet 1. Small RNA libraries or miRNA genome wide identifications published between 2008 and 2012 in model legumes. Mtr (*Medicago truncatula*); Gma (*Glycine max*); Lja (*Lotus japonicus*)
