# Supplementary material for: Small RNA pathways and diversity in model legumes: lessons from genomics
Source: Front Plant Sci. 2013 Jul 10;4:236. doi: 10.3389/fpls.2013.00236 (PMC3707012; doi:10.3389/fpls.2013.00236)
Supplement: Supplementary file 2 [file DataSheet2.PDF]

miRNA name	Organism	Reference	Role in nodulation
miR164	*Medicato truncatula*	D'haeseleer *et al*., 2011	Nodule organogenesis
miR166	*M. truncatula*	Boualem *et al*., 2008	Lateral root and nodule formation. Root vascular tissues differentiation
miR169	*M. truncatula*	Combier *et al*., 2006	Nodule differentiation
miR171h	*M. truncatula*	Lauressergues *et al*., 2012	Nodule and mycorrhiza establishment
miR482	*Glycine max*	Li *et al*., 2010	Nodule number
miR1507	*G. max*	Subramanian *et al*., 2008	Highly expressed in roots inoculated by symbiotic bacteria. Unknown function
miR1511	*G. max*	Li *et al*., 2010	Nodule number
miR1512	*G. max*	Li *et al*., 2010	Nodule number
miR1515	*G. max*	Li *et al*., 2010	Nodule number
miR1521	*G. max*	Subramanian *et al*., 2008	Highly expressed in roots inoculated by symbiotic bacteria. Unknown function
miR4416a	*G. max*	Turner *et al*., 2012	Highly expressed in nodules. Unknown function
miR4416b	*G. max*	Turner *et al*., 2012	Highly expressed in nodules. Unknown function
miR13587	*G. max*	Turner *et al*., 2012	Highly expressed in nodules. Unknown function
miR50841	*G. max*	Turner *et al*., 2012	Highly expressed in nodules. Unknown function
miR397	*Lotus japonicus*	de Luis *et al*., 2012	Nodule establishment and maintenance . Copper homeostasis in nodules
miR398	*L. japonicus*	de Luis *et al*., 2012	Copper homeostasis in nodules
miR171c	*L. japonicus*	de Luis *et al*., 2012	Nodule establishment and maintenance

Data Sheet 2. Reported miRNAs with a putative function in nodulation.
