# Supplementary material for: Small RNA pathways and diversity in model legumes: lessons from genomics
Source: Front Plant Sci. 2013 Jul 10;4:236. doi: 10.3389/fpls.2013.00236 (PMC3707012; doi:10.3389/fpls.2013.00236)
Supplement: Supplementary file 3 [file DataSheet3.PDF]

Species	Gene accession	Protein name	AtDCL best homologue	Protein length (aminoacids)	Molecular Weight (KDa)	Reference	Previous gene ID
**DICER-LIKE**							
Oryza sativa	LOC_Os03g02970	OsDCL1a	AtDCL1	1884	210	Kapoor et al., 2008	
	LOC_Os03g38740	OsDCL2a	AtDCL2	1411	158	Kapoor et al., 2008	
	LOC_Os09g14610	OsDCL2b	AtDCL2	1377	155	Kapoor et al., 2008	*not complete.
	LOC_Os01g68120	OsDCL3a	AtDCL3	1598	1598	Kapoor et al., 2008	
	LOC_Os10g34430	OsDCL3b	AtDCL3	1572	1572	Kapoor et al., 2008	
	LOC_Os04g43050	SHOI	AtDCL4	1059	1059	Kapoor et al., 2008	
Arabidopsis thaliana	At1g01040	AtDCL1		1909	214		
	At3g03300	AtDCL2		1388	157		
	At3g43920	AtDCL3		1580	177		
	At5g20320	AtDCL4		1702	191		
Populus trichocarpa	Potri.002G181400.1	PtDCL902	AtDCL1	1967	221	Margis et al., 2006	Pt2g14226280
	Potri.010G181400.1	PtDCL901	AtDCL2	1354	153	Margis et al., 2006	Pt8g4686890
	Potri.008G075900.1	PtDCL903	AtDCL2	1456	164	Margis et al., 2006	Pt10g16358340
	Potri.018G047500.1	PtDCL904	AtDCL3	1624	181	Margis et al., 2006	Pt18g3481550
	Potri.006G188800.1	PtDCL905	AtDCL4	1638	184	Margis et al., 2006	Pt6g11470720
Glycine max	Glyma03g42290	GmDCL1a	AtDCL1	1913	215	Curtin et al., 2011	
	Glyma19g45060	GmDCL1b	AtDCL1	1902	212	Curtin et al., 2011	
	Glyma09g02930	GmDCL2a	AtDCL2	1414	160		
	Glyma09g02920	GmDCL2b	AtDCL2	1305	148		
	Glyma04g06060	GmDCL3	AtDCL3	1530	172		
	Glyma17g11240	GmDCL4a	AtDCL4	1679	190	Curtin et al., 2011	
	Glyma13g22450	GmDCL4b	AtDCL4	1394	157	Curtin et al., 2011	
Medicago truncatula	Medtr7g118350	MtDCL1	AtDCL1	1971	222	Capitao et al.,2011	Medtr7g146220
	Medtr2g030490	MtDCL2	AtDCL2	1418	161	Capitao et al., 2011	Medtr2g129960
	Medtr3g105390	MtDCL3	AtDCL3	1758	196	Capitao et al., 2011	Medtr3g139020
Lotus japonicus	chr1.CM0105.1760.r2.m	LjDCL1	AtDCL1	1945	217		
	chr6.CM0437.480.r2.m	LjDCL2a	AtDCL2	1369	155		

	chr6.CM0437.490.r2.m	LjDCL2b	AtDCL2	1331	151		
	chr1.CM0133.1260.r2.m	LjDCL3	AtDCL3	1356	151		
	chr1.CM0133.1260.r2.m	LjDCL4	AtDCL4	1419	161		
**ARGONAUTE**							
Oryza sativa	LOC_Os02g45070	OsAGO1a	AtAGO10	1083	120	Kapoor et al., 2008	
	LOC_Os04g47870	OsAGO1b	AtAGO10	1102	122	Kapoor et al., 2008	
	LOC_Os02g58490	OsAGO1c	AtAGO10	1012	113	Kapoor et al., 2008	
	LOC_Os06g51310	OsAGO1d	AtAGO10	1039	116	Kapoor et al., 2008	
	LOC_Os04g52540	OsAGO2	AtAGO2	1038	111	Kapoor et al., 2008	
	LOC_Os04g06770	OsAGO4b	AtAGO4	912	102	Kapoor et al., 2008	
	LOC_Os01g16870	OsAGO15	AtAGO6	2237	147	Kapoor et al., 2008	
	LOC_Os03g58600	OsMELI	AtAGO5	1059	117	Kapoor et al., 2008	
	LOC_Os03g33650	OsSHL4	AtAGO7	1055	118	Kapoor et al., 2008	
	LOC_Os06g39640	OsPNHI	AtAGO10	974	108	Kapoor et al., 2008	
	LOC_Os04g52550	OsAGO3	AtAGO3	1110	123	Kapoor et al., 2008	
	LOC_Os07g09020	OsAGO14	AtAGO5	1053	114	Kapoor et al., 2008	
	LOC_Os03g57560	OsAGO13	AtAGO5	1061	121	Kapoor et al., 2008	
	LOC_Os07g16224	OsAGO16	AtAGO6	364	40.5	Kapoor et al., 2008	
	LOC_Os02g07310	OsAGO17	AtAGO10	877	98.9	Kapoor et al., 2008	
	LOC_Os03g47820	OsAGO12	AtAGO6	977	107	Kapoor et al., 2008	
	LOC_Os03g47830	OsAGO11	AtAGO6	895	99.8	Kapoor et al., 2008	
	LOC_Os07g28850	OsAGO18	AtAGO7	1089	118	Kapoor et al., 2008	
	LOC_Os01g16850	OsAGO4a	AtAGO4	905	101	Kapoor et al., 2008	
Arabidopsis thaliana	At1g48410	AtAGO1		1048	116		
	At1g31280	AtAGO2		1014	113		
	At1g31290	AtAGO3		1194	129		
	At2g27040	AtAGO4		924	103		
	At2g27880	AtAGO5		997	111		
	At2g32940	AtAGO6		878	987		
	At1g69440	AtAGO7		990	113		

	At5g21030	AtAGO8		850	955		
	At5g21150	AtAGO9		896	101		
	At5g43810	AtAGO10		988	111		
	Potri.010g163800	PtAGO7	AtAGO7	1030	117		
Populus trichocarpa	Potri.012g037100	PtAGO1a	AtAGO1	1062	118		
	Potri.015g029000	PtAGO1b	AtAGO1	875	98.7		
	Potri.015g117400	PtAGO2a	AtAGO2	895	101		
	Potri.012g118700	PtAGO2b	AtAGO2	1039	114		
	Potri.006g025900	PtAGO4a	AtAGO4	930	104		
	Potri.016g024200	PtAGO4b	AtAGO4	921	103		
	Potri.008g010500	PtAGO4c	AtAGO4	923	103		
	Potri.006g118600	PtAGO5a	AtAGO5	879	98.0		
	Potri.001g213700	PtAGO5b	AtAGO5	985	110		
	Potri.009g001500	PtAGO5c	AtAGO5	987	110		
	Potri.014g159400	PtAGO6	AtAGO6	910	102		
	Potri.008g158800	PtAGO10a	AtAGO10	996	112		
	Potri.010g081300	PtAGO10b	AtAGO10	999	112		
Glycine max	Glyma16g34300	GmAGO1a	AtAGO1	1052	116		
	Glyma09g29720	GmAGO1b	AtAGO1	1071	119		
	Glyma15g13260	GmAGO2b	AtAGO2	949	108		
	Glyma20g02820	GmAGO2a	AtAGO2	982	110		
	Glyma02g44260	GmAGO4a	AtAGO4	906	101		
	Glyma20g12070	GmAGO4b	AtAGO4	915	102		
	Glyma14g04510	GmAGO4c	AtAGO4	906	101		
	Glyma12g08860	GmAGO5a	AtAGO5	921	104		
	Glyma11g19650	GmAGO5b	AtAGO5	723	81.9		
	Glyma13g26240	GmAGO6	AtAGO6	913	102		
	Glyma02g12430	GmAGO7	AtAGO7	762	87.2		
	Glyma06g47230	GmAGO9	AtAGO9	873	98.9		
	Glyma10g38770	GmAGO10a	AtAGO10	973	110		
	Glyma02g00510	GmAGO10b	AtAGO10	972	109		
	Glyma10g00530	GmAGO10c	AtAGO10	445	49.8		

	Glyma20g28970	GmAGO10d	AtAGO10	927	104		
	Glyma17g12850	GmAGO12a	AtAGO10	903	102		
	Glyma05g08170	GmAGO12b	AtAGO10	729	82.3		
	Glyma04g21450	GmAGO12c	AtAGO10	671	76.0		
	Glyma06g23920	GmAGO12d	AtAGO10	909	103		
Medicago truncatula	Medtr4g083610	MtAGO2a	AtAGO2	916	104	Capitao et al., 2011	Medtr4g114860
	Medtr2g028910	MtAGO2b	AtAGO2	883	101	Capitao et al., 2011	Medtr2g04460
	Medtr3g078660	MtAGO4a	AtAGO4	824	92.4	Capitao et al., 2011	Medtr3g111450
	Medtr5g087870.1	MtAGO4b	AtAGO4	942	705	Capitao et al., 2011	Medtr5g094930
	Medtr5g087890	MtAGO4c	AtAGO4	912	103	Capitao et al., 2011	Medtr5g094940
	Medtr1g106830	MtAGO4d	AtAGO4	902	102		
	Medtr3g083300	MtAGO6	AtAGO6	935	105	Capitao et al., 2011	Medtr3g105930
	Medtr3g010650	MtAGO11a	AtAGO4	876	99.9	Capitao et al., 2011	Medtr3g016400<br>Medtr3g016420
	Medtr5g087870.3	MtAGO11b	AtAGO4	908	101	Capitao et al., 2011	Medtr3g16410
	Medtr5g042590	MtAGO7	AtAGO7	1016	117	Capitao et al., 2011	Medtr5g045600
	Medtr4g113200	MtAGO12a/b	AtAGO10	876	98.6	Capitao et al., 2011	Medtr8g118920
	Medtr2g059590	MtAGO12c	AtAGO10	520	59.6	Capitao et al., 2011	Medtr2g074570
Lotus japonicus	chr2.CM0435.710.r2.m	LjAGO1	AtAGO10	1076	118		
	chr4.CM0229.30.r2.m	LjAGO2a	AtAGO2	1101	120		
	chr6.CM0066.170.r2.a	LjAGO2b	AtAGO2	997	113		
	chr2.CM0031.300.r2.m	LjAGO4b	AtAGO4	927	103		
	chr6.CM1650.210.r2.m	LjAGO4a	AtAGO4	921	103		
	chr3.CM0396.310.r2.d	LjAGO5	AtAGO5	970	109		
	CM1092.250.r2.m	LjAGO6	AtAGO6	905	101		
	chr2.CM0608.180.r2.m	LjAGO7	AtAGO7	1020	117		
	chr5.CM0200.560.r2.a	LjAGO10	AtAGO10	982	110		

Data Sheet 3. Characteristics of DICER and ARGONAUTE proteins in model legumes, rice, *Arabidopsis thaliana* and poplar. For *Arabidopsis thaliana*, rice (*O. sativa)* and poplar (*P. trichocarpa)*, gene accessions and sequences were retrieved from Genbank (NCBI), except for *P. trichocarpa* AGO proteins that were searched by tBLASTX in, http:://www.phytozome.net/poplar (Tuskan et al., 2006; *Populus*

trichocarpa v3.0, DOE-JGI). For model legumes, DCL and AGO genes were found by searching homologues of *Arabidopsis thaliana* genes by tBLASTX in available genomic databases: *Lotus japonicus* (Miyakogusa.jp 2.5, Sato et al., 2008), *Medicago truncatula* (Mt 3.5.1, Young et al., 2011), and *Glycine max* (Glyma1.181, http://www.plantgdb.org/GmGDB/), and named according to their similarity with *A. thaliana* proteins or according to previous publications (referenced in the last column). Protein length (in aminoacids) and molecular weight (KDa) were calculated with PROTEIN CALCULATOR v3.3 (http://www.scripps.edu/~cdputnam/protcalc.html).
