# Supplementary material for: Small RNA pathways and diversity in model legumes: lessons from genomics
Source: Front Plant Sci. 2013 Jul 10;4:236. doi: 10.3389/fpls.2013.00236 (PMC3707012; doi:10.3389/fpls.2013.00236)
Supplement: Supplementary file 4 [file DataSheet4.PDF]

Name	Gene	Domains			Protein length (aminoacids)
		DUF1785	PAZ	MID-PIWI	
GmAGO1a	Glyma16g34300	339-391	396-528	680-1000	1052
GmAGO1b	Glyma09g29720	352-404	409-541	693-1014	1071
GmAGO2b	Glyma15g13260	252-305	306-438	604-902	949
GmAGO2a	Glyma20g02820	279-332	337-480	633-932	982
GmAGO4c	Glyma14g04510	224-276	277-414	560-686	906
GmAGO4a	Glyma02g44260	224-276	277-414	560-868	906
GmAGO4b	Glyma20g12070	230-282	283-422	568-876	915
GmAGO5	Glyma12g08860	226-278	283-422	566-886	921
GmAGO6	Glyma13g26240	231-283	284-423	569-877	913
GmAGO7	Glyma02g12430	64-116	124-255	412-723	762
GmAGO9	Glyma06g47230	185-237	238-374	523-834	873
GmAGO10a	Glyma10g38770	266-318	323-455	607-928	973
GmAGO10d	Glyma20g28970	220-272	277-409	561-882	927
GmAGO10b	Glyma02g00510	267-319	324-456	608-929	972
GmAGO12a	Glyma17g12850	205-257	262-394	546-867	903
GmAGO5b	Glyma11g19650	96-148	153-292	436-721	723
GmAGO12b	Glyma05g08170	210-262	267-399	528-729	729
GmAGO10c	Glyma10g00530			85-406	445
GmAGO12c	Glyma04g21450	211-263	268-400	552-662*	671
GmAGO12d	Glyma06g23920	211-263	268-400	552-87	909
MtAGO12a/b	Medtr4g113200.1	178-230	235-367	519-840	876
MtAGO12c	Medtr2g059590.1	203-255	260-392		520
MtAGO2a	Medtr4g083610.1	274-327	328-475	628-927	916
MtAGO2b	Medtr2g028910.1	339-392	393-526	693-991	883
MtAGO4a	Medtr3g078660.1			1-269	824
MtAGO4b	Medtr5g087870.1	262-314	315-454	600-908	942
MtAGO11b	Medtr5g087870.3	222-274	275-414	560-868	908
MtAGO4d	Medtr1g106830.1	221-273	274-412	558-865	902
MtAGO4c	Medtr5g087890.1	244-296	297-441	585-893	912
MtAGO11a	Medtr3g010650.1	192-244	245-382	53-841	876
MtAGO6	Medtr3g083300.1	256-308	309-449	595-911	935
MtAGO7	Medtr5g042590.1	316-368	376-516	664-977	1016
LjAGO1	chr2.CM0435.710.r2.m	365-417	422-554	706-1027	1076
LjAGO2a	chr4.CM0229.30.r2.m	397-450	451-583	752-1051	1101
LjAGO2b	chr6.CM0066.170.r2.a	306-359	360-498	660-958	997
LjAGO4b	chr2.CM0031.300.r2.m	238-290	291-432	579-887	927
LjAGO4a	chr6.CM1650.210.r2.m	238-290	291-427	574-882	921
LjAGO5	chr3.CM0396.310.r2.d	270-322	327-458	611-932	970
LjAGO6	CM1092.250.r2.m	222-274	275-414	561-869	905
LjAGO7	chr2.CM0608.180.r2.m	321-373	381-512	669-981	1020
LjAGO10	chr5.CM0200.560.r2.a	274-326	331-463	615-936	982

Data Sheet 4. Conserved domain composition of legume AGO proteins. For all predicted ARGONAUTES of *G. max (Gm)*, *M. truncatula (Mt)*, and *L. japonicus (Lj)*, gene name and accessions, protein length (aminoacid number) and positions of the DUF1785, PAZ and MID_PIWI conserved domains on the proteins (according Simple Modular Architecture Research Tool-SMART version 7, Letuvic et al., 2012) are indicated.
