# Supplementary material for: Small RNA pathways and diversity in model legumes: lessons from genomics
Source: Front Plant Sci. 2013 Jul 10;4:236. doi: 10.3389/fpls.2013.00236 (PMC3707012; doi:10.3389/fpls.2013.00236)
Supplement: Supplementary file 5 [file DataSheet5.PDF]

```
LjAGO2a         --FLLCVMAGKDQGYKCLKWIAETKVGLVTQCCLSGNANEGSDQYLTNLALKINAKIGGT
GmAGO2a         --FLLCVMSDKHQGYKCLKWIAETKVGIVTQCCLSGIANEGKDQYLTNLALKINAKIGGS
MtAGO2a         --FLLCVMANKDPGYKSLKWIAETKVGIVTQCCLSGNANEGKDQYLTNLALKINAKIGGS
LjAGO2b         --FLLCVMAKKSSGYKYLKWISETKIGIVTQCCLSSSANEGEDKFYTNLALKINAKLGGS
GmAGO2b         --FLLCVMAKKSPGYKYLKWISETKLGILTQCCLSNSANEGEDKFYTNLALKINAKLGGS
MtAGO2b         --FLLCVMANKSPGYKYLKWISETKVGIVTQCCLSYSANQGDDKFYTYLALKINAKLGGS
LjAGO7          --LLICVMERKHKGYADLKRIAETSIGLISQCCLYPNLCKLSSQFLANLALKINAKVGGC
GmAGO7          --LLICIMERKHKGYADLKRIAETSVGVVSQCCLYPNLNKLSSQFLANLALKINAKVGGC
MtAGO7          --LLICIMEKKHKGYADLKRIAETSVGVVSQCCLYPNLIKLSSQFLANLALKINAKVGGC
GmAGO12b        -LLIAILPDNNGSLYGDLKRICETDLGLISQCCLTKHVFKINRQYLANVALKINVKMGGR
GmAGO12c        -LLIAILPDNNGSLYGDLKRICETDLGLISQCCLTKHVFKINRQYLANVALKINVKMGGR
GmAGO12a        -LLIAILPDNNGSLYGDLKRICETDLGLISQCCLTKHVFKINRQYLANVALKINVKMGGR
GmAGO12d        -LLIALLPDNNGSLYGDLKRICETDLGLISQCCLTKHVFKINRQYLANVALKINVKMGGR
MtAGO12a/b      -LVVAILPDNNGSLYGDLKKICETDLGLISQCCLTKYVFKINRQYLSNVALKINVKMGGR
GmAGO10b        -LLLAILPDNNGSLYGDLKRICETDLGLISQCCLTKHVFKITKQYLANVSLKINVKMGGR
GmAGO10c        -LLLAILPDNNGSLYGDLKRICETDLGLISQCCLTKHVFKITKQYLANVSLKINVKMGGR
GmAGO10a        -LLLAILPDNNGSLYGDLKRICETDLGLISQCCLTKHVFKITKQYLANVSLKINVKMGGR
GmAGO10d        -LLLAILPDNNGSLYGDLKRICETDLGLISQCCLTKHVFKITKQYLANVSLKINVKMGGR
LjAGO10         -LLLAILPDNNGSLYGDLKRICETDLGLISQCCLTKHVFKITKQYLANVSLKINVKMGGR
GmAGO1a         -LLIVILPDNNGSLYGDLKRICETDLGLVSQCCLTKHVFKMSKQYLANVALKINVKVGGR
GmAGO1b         -LLIVILPDNNGSLYGDLKRICETDLGLVSQCCLTKHVFKMSKQYLANVALKINVKVGGR
LjAGO1          -LLIVILPDNNGSLYGDLKRICETDLGLVSQCCLTKHVFKMSKQYLANVALKINVKVGGR
GmAGO5          -LLIIILPDFEG-SYEKIKRICETELGIVSQCCQPRHVCQMKPQYLENVALKINVKVGGS
GmAGO5b         -LLIIILPDLKG-SYGKIKRICETELGIVSQCCLPRHVYQMKPQYLENVALKINVKVGGS
LjAGO5          -LLIIILPDVKG-SYGKIKKICETELGIVSQCCQPRQVQKLNKQYLENLALKINVKVGGR
LjAGO4a         FLLCLLPDRKNCEIYGPWKKKNLADFGIVNQCMCP---LRVNDQYLTNIMLKINAKLGGL
GmAGO4b         FLLCLLPDRKNCDIYGPWKKKNLADFGIINQCMCP---LRVNDQYLTNVMLKINAKLGGL
MtAGO4a         -------------------------------------------MMFYPFNLSIVLQLGGL
MtAGO4b         FLLCLLSERKNSDLYGPWKKKNLAEFGIVTQCIAP---TRVNDQYLTNVLLKINAKLGGM
MtAGO11b        FLLCLLSERKNSDLYGPWKKKNLAEFGIVTQCIAP---TRVNDQYLTNVLLKINAKLGGM
MtAGO4c         FLLCLLPERKNSDLYGPWKKKNLAEFGIVTQCIAP---TRVNDQYLTNVLLKINAKLGGM
GmAGO4c         FLLCLLPERKNSDLYGPWKKKNLAEFGIVTQCIAP---TRVNDQYLTNVLLKINAKLGGL
GmAGO4a         FLLCLLPERKNSDLYGPWKKKNLAEFGIVTQCIAP---TRVNDQYLTNVLLKINAKLGGL
LjAGO4b         FLLCLLPERKNSDLYGPWKKKNLAEYGIVTQCISP---TRVNDQYLTNVLMKINAKLGGL
MtAGO4d         -FILCLLPQKNCDLYGPWKKKNLAEEGIITQCIVP---SKVNDQYLTNVLLKINAKLDGI
GmAGO9          FLLCILPEKKNSDIYGPWKKKSLVEEGIVTQCIAP---TKINDQYITNVLLKINAKYGGM
MtAGO11a        LLLCILPVSRNCNIYGPWKRRCLVDEGIATQCIAP---TKINDHYIINVLLKINAKLGGM
LjAGO6          LILCVLPEKKNCDIYGPWKKKCLSEFGVVTQCISP---LKITDQYLTNVLLKINSKLGGI
GmAGO6          LILCVLPERKICDIYGPWKKKCLSEIGVVTQCIAP---VKITNQYLTNVLLKINSKLGGI
MtAGO6          LILCVLPERKNCDIYGPWKRKCLSDVGVVTQCISP---LKITDQYLTNVLLKINSKLGGI

LjAGO2a         NVELVNRLP-----HIDGEGDVMFIGADVNHPGSRDINSPSIAAVVATVNWPAANRYAAR
GmAGO2a         NVELINRLP-----HFEGEGHVMFIGADVNHPASRDINSPSIAAVVATVNWPAANRYAAR
MtAGO2a         NVELINRLP-----HFEDESHVMFIGADVNHPGSRDTNSPSIVAVVATTNWPAANRYAAR
LjAGO2b         NVELSNRLP-----YFEGEGHVMFVGADVNHPGSRDTRSPSIAAVVATVNWPAANRYAAR
GmAGO2b         NVELSNGLP-----YFEDEGDVMFLGADVNHPGYQDTRSPSIAAVVATVNWPAANRYAAR
MtAGO2b         NVELNNRLP-----YFEGEEHVMFIGADVNHPGSRDNKSPSIVAVVATINWPAANRYAAR
LjAGO7          TVALYNSLPSQLPRLFHIDEPVIFMGADVTHPHPLDDSSPSVAAVVGSMNWPTANKYISR
GmAGO7          TVALYNSLPSQLPRLFHIDEPVIFMGADVTHPHPLDDVSPSVAAVVGSMNWPTANKYISR
MtAGO7          TVALYNSLPSQLPRLFNIDEPVMFMGADVTHPHPLDDSSPSVAAVVGSMNWPTANKYISR
GmAGO12b        NTVLLDALSWRIP--LVSDIPTIIFGADVTHPESGEDPCPSIAAVVASQDWPEVTKYAGL
GmAGO12c        NTVLLDALSWRIP--LVSDIPTIIFGADVTHPESGEDSCPSIAAVSISKLIYTT------
GmAGO12a        NTVLLDALSWRIP--LVSDIPTIIFGADVTHPESGEDPCPSIAAVVASQDWPEVTKYAGL
GmAGO12d        NTVLLDALSWRIP--LVSDIPTIIFGADVTHPESGEDSCPSIAAVVASQDWPEVTKYAGL
MtAGO12a/b      NTVLLDAISCRIP--LVSDVPTIIFGADVSHPESGEDVCPSIAAVVASQDWPEVTKYAGL
GmAGO10b        NTVLVDAVSCRIP--LVSDIPTIIFGADVTHPENGEDSSPSIAAVVASQDWPEVTKYAGL
GmAGO10c        NTVLVDAVSCRIP--LVSDIPTIIFGADVTHPENGEDSSPSIAAVVASQDWPELTKYAGL
GmAGO10a        NTVLLDAVSSRIP--LVSDMPTIIFGADVTHPENGEELSPSIAAVVASQDWPEVTKYAGL
GmAGO10d        NTVLLDAVSCRIP--LVSDIPTIIFGADVTHPENGEDSSPSIAAVVASQDWPEVTKYAGL
LjAGO10         NTVLLDALSCRIP--LVSDIPTIIFGADVTHPENGEDSSPSIAAVVASQDWPEVTKYAGL
GmAGO1a         NTVLVDALSRRIP--LVSDRPTIIFGADVTHPHPGEDSSPSIAAVVASQDYPEITKYAGL
GmAGO1b         NTVLVDALSRRIP--LVSDRPTIIFGADVTHPHPGEDSSPSIAAVVASQDYPEITKYAGL
LjAGO1          NTVLVDALSRRIP--LVSDRPTIIFGADVTHPHPGEDSSPSIAAVVASQDYPEITKYAGL
GmAGO5          NTVLNDAIARIIP--RVSDRPTLILGADVTHPQPGEDSSPSIAAVVASMDWPYVTRYRGV
GmAGO5b         NTVLNDAFTRRIP--HVSDLPTIILGADVTHPQPGEDYSPSIAAVVASMDWPYVTKYRGV
LjAGO5          NTVLSDAFDRRIP--HVSDKHTIIFGADVTHPQPGEDSSPSIAAVVASMDWPWVTKYKGT
LjAGO4a         NSLLSVELSPSLP--IVSKAPTLILGMDVSHGSPGQTDIPSIAAVVSSREWPLISKYRAC
GmAGO4b         NSLLGVEHSPSLP--VVSKAPTLILGMDVSHGSPGQTDIPSIAAVVSSRHWPLISKYRAC
```

```
MtAGO4a         NSLLGVESSPSLP--IVSKAPTLILGMDVSHGSPGQTDIPSIAAVVSSRQWPLISKYRAC
MtAGO4b         NSLLGVEHSPSIP--IVSKAPTLILGMDVSHGSPGQTEIPSIAAVVSSRQWPLISKYRAC
MtAGO11b        NSLLGVEHSPSIP--IVSKAPTLILGMDVSHGSPGQTEIPSIAAVVSSRQWPLISKYRAC
MtAGO4c         NSWLGVEHSRSIP--IVSKVPTLILGMDVSHGSPGQPDIPSIAAVVSSRKWPLISKYRAC
GmAGO4c         NSMLGVEHSPSIP--IVSRAPTIIIGMDVSHGSPGQTDIPSIAAVVSSREWPLISKYRAS
GmAGO4a         NSILGVEHSPSIP--IVSRAPTIIIGMDVSHGSPGQTDIPSIAAVVSSREWPLISKYRAS
LjAGO4b         NSVLGVEMNPSIP--IVSKVPTIILGMDVSHGSPGQSDIPSIAAVVSSREWPLISKYRAC
MtAGO4d         NSFLGIEHARSMP--IVSREPTLILGMDVSHGSPGQSEIPSIAAVVSSRQWPLISKYRAC
GmAGO9          NSYLSVELCNSIP--FVSAVPTLILGMDVSHGSPGRSDVPSIAAVVSSRCWPQISRYRAS
MtAGO11a        NSFLLTEFKHSIP--LFSKIPTLVIGMDVSHGSQGQSEALSIAAVVSSRCWPQISRYKAV
LjAGO6          NSLLTIELSGNLP--LIEDTPTMILGMDVSHSSPGQSDVPSIAAVVGSRLWPLISRYRAS
GmAGO6          NSLLAIEHSGHLP--LIKDTPTMILGMDVSHNSLGRLDSPSIAAVVGSRHWPLISRYRAS
MtAGO6          NSLLAIEHSGHLP--LIKDTPTMILGMDVSHGSPGRSDIPSIAAVVGSRCWPLISRYRAS

LjAGO2a         VCAQGHRVEKIVNFG------------------EVCLDLVTYYESLNKVRPEKIVIFRDG
GmAGO2a         VCAQGHRVEKILNFG------------------RICYELVSYYDRLNKVRPEKIVVFRDG
MtAGO2a         VCAQEHCTEKILNFG------------------EICLDLVRHYEKLNKVRPQKIVIFRDG
LjAGO2b         VCPQYNRCEKILNFG------------------EVCLELVTCYCRINGVRPERIVVFRDG
GmAGO2b         VFPQYNRSEKILNFG------------------DVCLELVACYRRMNGVRPERIVIFRDG
MtAGO2b         VCPQFNRSEKILNFG------------------EICVELVSCYWQKNGVRPEKIVVFRDG
LjAGO7          IRSQTHRQEIIQDLG------------------PMVGELLDDFYQEVEKLPNRIVFFRDG
GmAGO7          IRSQTHRQEIILDLG------------------AMVGELLDDFYQEVEKLPNRIIFFRDG
MtAGO7          IRSQTHRQEIIADLG------------------AMVGELLEDFYQEVEKLPNRIIFFRDG
GmAGO12b        VCAQPHREELIQDLFKCWKNPHHG----IVYG----------------------GMIRDG
GmAGO12c        ------------------------------------------------------------
GmAGO12a        VCAQPHREELIQDLFKCWKDPHHG----IVYGGMIRELLLSFKKA-TGQKPLRIIFYRDG
GmAGO12d        VCAQPHREELIQDLFRCWKDPQRG----VMYGGMIRELLLSFKKA-TGQKPLRIIFYRDG
MtAGO12a/b      VCAQPPREEIIKDLFKCWNDPRRG----IVYGGMIRELLLSFQKA-TGKKPCRILFYRDG
GmAGO10b        VCAQAHRQELIQDLYKMWHDPVRG----LVSGGMIRDLLISFRKA-TGQKPLRIIFYRDG
GmAGO10c        VCAQAHRQELIQDLYKMWHDPVRG----LVSGGMIRDLLISFRKA-TGQKPLRIIFYRDG
GmAGO10a        VCAQAHRQELIQDLYKTWQDPVRG----TVSGGMIRDLLVSFRKA-TGQKPLRIIFYRDG
GmAGO10d        VCAQAHRQELIQDLYKTWQDPVRG----TVSGGMIRDLLVSFRKA-TGQKPLRIIFYRDG
LjAGO10         VCAQAHRQELIQDLYKTWQDPVRG----TVSGGMIRDLLVSFRKA-TGQKPLRIIFYRDG
GmAGO1a         VCAQVHRQELIQDLFKQWQDPVRG----TVTGGMIKELLISFRRA-TGQKPQRIIFYRDG
GmAGO1b         VCAQAHRQELIQDLFKQWQDPVRG----TVTGGMIKELLISFRRA-TGQKPQRIIFYRDG
LjAGO1          VCAQAHRQELIQDLFKQWQDPVRG----TLTGGMIKELLISFRRA-TGQKPQRIIFYRDG
GmAGO5          VSAQTHREEIIQDLYNTCEDPVKG----KVHSGIIRELLRAFRLS-TNQKPERIIFYRDG
GmAGO5b         VSAQTHREEIIQDLYNTHEDPVRG----KTHSGIIRELLRAFRLS-TKTKPERIIFYRDG
LjAGO5          VSAQAHREEIIQDLFTTFEDPKRG----LVQGGIIRELIRSFYIANGKRKPERIIFYRDG
LjAGO4a         VRTQSAKVEMIDNLFKQVSEK--------EDEGIMRELLLDFYLSSGKRKPDNIIIFRDG
GmAGO4b         VRTQSAKMEMIDNLFKLVSEK--------EDEGIIRELLLDFYTTSGRRKPENIIIFRDG
MtAGO4a         VRTQSAKVEMIDNLFKKVSDT--------EDEGIMRELLLDFYTSSKNRKPDNIIIFRDG
MtAGO4b         VRTQGAKVEMIDNLFKPVSDT--------EDEGIIRELLIDFYNSSGNRKPDNIIIFRDG
MtAGO11b        VRTQGAKVEMIDNLFKPVSDT--------EDE----------------------GIIRDG
MtAGO4c         VRTQGSKVEMIDNLFKPVSDK--------EDEGIIRELLLDFFHSSEERRPENIIIFRDG
GmAGO4c         VRTQSPKMEMIDNLFKKVSDK--------EDEGIMRELLLDFYTSSGNRKPDNIIIFRDG
GmAGO4a         VRTQSPKMEMIDNLFKKVSDK--------EDEGIMRELLLDFYTSSGNRKPDNIIIFRDG
LjAGO4b         VRTQSPKVEMIDNLFKQVSEK--------EDEGIIRELLIDFYSSSGKRKPDNIIIFRDG
MtAGO4d         VRTQGSKVEMIDNLFKPMPNN--------EDAGIIRELLVDFYNSSGQRKPANIIIFRDG
GmAGO9          VRTQSSKVEMIQSLFKPVANTN-------KDEGIIREVLLDFEITSFKRKPQQIIIFRDG
MtAGO11a        VRTQSSKVEIVQSLFKPVSDT--------KDDGIISELLKDFQTTSG-VKPQQIIIFRDG
LjAGO6          VRTQSSKVEIIDALYKPLDGG--------KDDGIIRELLLDFYESSNGRKPAQIIVFRDG
GmAGO6          VRMQASKVEMIDALYKPLENG--------SDDGIIRELLLDFYDSSNGRKPTQFIVFRDG
MtAGO6          VRSQSPKVEMIDSLFKLVDKMNDDGILEKKDDGIIRELLLDFYSSSGNRRPTQIILFRDG


LjAGO2a         VSESQFLMVLTEELQDLRRAFSR--SNYFPTITLIVAQKRHQTRLFPASAKDGAPSGN--
GmAGO2a         VSESQFHMVLTEELQDLKSVFSD--ANYFPTITIIVAQKRHQTRFFPVGPKDGIQNGN--
MtAGO2a         VSESQFHMVLGEELKDLKTVFQH--SNYFPTITLIVAQKRHQTRLFPAGVREGAPSGN--
LjAGO2b         VSEYQFDMVLNEELLDLKKAFQR--LNYFPTITLIVAQKRHHTRFFPEGWRDGSSSGN--
GmAGO2b         VSEYQFDMVLNEELLDLKGVFQR--VNYFPTITLIVTQKRHHTRFFPEGWRDGSSSGN--
MtAGO2b         VSEFQFDMVLNEELLDLKRAFQR--LNYFPTITLIVAQKRHQTRFFPDSWRDGSSSGN--
LjAGO7          VSETQFHKVMQEELQSIRHACER-FPDYKPLITFAVVQKRHHTRLFPFPGETDPSSPQNN
GmAGO7          VSETQFYKVLEEELQSIRCACSR-FPGYKPTITFAVVQKRHHTRLFPFETDQS-STQKNN
MtAGO7          VSETQFYKVLQEELQSIKQACSSRFHGYKPFITFVVVQKRHHTRLFPADTDQSSMHNNFH
GmAGO12b        VSEGQFYQVLLHELDAIRKGTS--------------------------------------
GmAGO12c        ------------------------------------------------------------
GmAGO12a        VSEGQFYQVLLYELDAIRKACASLEPSYQPPVTFVVVQKRHHTRLFSNNHDDRNSTDKSG
```

```
GmAGO12d        VSEGQFYQVLLYELDAIRKACASLEPSYQPPVTFVIVQKRHHTRLFANNHDDRNSTDKSG
MtAGO12a/b      VSEGQFYQVLLYELDAIRKACASLEPGYQPPVTFVVVQKRHHTRLFSDNHNDRNSMDRSG
GmAGO10b        VSEGQFYQVLLYELDAIRKACASLEPNYQPPVTFIVVQKRHHTRLFANNHRDRNSTDKSG
GmAGO10c        VSEGQFYQVLLYELDAIRKACASLEPNYQPPVTFIVVQKRHHTRLFANNHRDRNSTDKSG
GmAGO10a        VSEGQFYQVLLYELDAIRKACASLEPNYQPPVTFIVVQKRHHTRLFANNYRDRSSTDRSG
GmAGO10d        VSEGQFYQVLLYELDAIRKACASLEPNYQPPVTFIVVQKRHHTRLFANNYRDRSSTDRSG
LjAGO10         VSEGQFYQVLLYELDAIRKACASLEPNYQPPVTFIVVQKRHHTRLFPNNHKDRSSTDKSG
GmAGO1a         VSEGQFYQVLLFELDAIRKACASLEPNYQPPVTFVVVQKRHHTRLFASNHHDKSSVDKSG
GmAGO1b         VSEGQFYQVLLFELDAIRKACASLEPNYQPPVTFVVVQKRHHTRLFASNHHDKSSFDRSG
LjAGO1          VSEGQFYQVLLFELDAIRKACASLEPNYQPPVTFVVVQKRHHTRLFASNHHDKSSVDRSG
GmAGO5          VSEGQFSQVLLYEMDAIRRACASLQEGYLPRVTFVVVQKRHHTRLFPVDHGSHDQTNKSG
GmAGO5b         VSEGQFSQVLLYEMDAIRRACASLQEDYMPRVTFVVVQKRHHTRLFPAEHGSRDQTDKSG
LjAGO5          VSEGQFSQVLLYEMDAIRKACMSLEDGYLPRVTFVVVQKRHHTRLFPADHRSRDQMDKSG
LjAGO4a         VSESQFNQVLNVELDQIMEACKFLDDKWEPKFVVIVAQKNHHTRFFQP------------
GmAGO4b         VSESQFNQVLNIELDRIIEACKFLDENWEPKFVVIVAQKNHHTRFFQP------------
MtAGO4a         VSESQFNQVLNIELDQIIEACKFLDENWTPKFVVIVAQKNHHTRFFQP------------
MtAGO4b         VSESQFNQVLNIELSQIIEACKFLDEKWNPKFLVIVAQKNHHTKFFQP------------
MtAGO11b        VSESQFNQVLNIELSQIIEACKFLDEKWNPKFLVIVAQKNHHTKFFQP------------
MtAGO4c         VSESQFNEVLNVELSQIIEACKFLDENWNPKFMVIVAQKNHHTKFFQP------------
GmAGO4c         VSESQFNQVLNIELDQIIEACKFLDEKWNPKFLVIVAQKNHHTKFFQP------------
GmAGO4a         VSESQFNQVLNIELDQIIEACKFLDEKWNPKFLVIVAQKNHHTKFFQP------------
LjAGO4b         VSESQFNQVLNIELNQIIEACKFLDETWNPKFLVIVAQKNHHTKFFQP------------
MtAGO4d         VSESQFNQVLNVELGQIIEACKFLDEDWNPKFLLIVAQKRHHTKFFQP------------
GmAGO9          VSESQFNQVLNIELSQIIEACKHLDEKWDPKFTLIIAQKNHHTRFFQANA----------
MtAGO11a        VSESQFNQVLNIELNEIIKACKCYDESWCPKFTLIVAQKNHHTRFFKAN-----------
LjAGO6          VSESQFNQVLDIEVNQIIKAYQHLGEVDVPKFTVIVAQKNHHIKLFQAN-----------
GmAGO6          VSESQFEQVLTIELNQIIKAYQHLGEVNVPQFTVIVAQK-KHIKLFLPN-----------
MtAGO6          VGESQFQHVLDIELNQIIKAYKHI-DGDVPKFTVIVAQKNHHTKLFQAN-----------


LjAGO2a         -----VLPGTVVDTIVVHPFEFDFYLCSHYGSLGTSKPTHYHVLWDEHKFSSDDLQKLIY
GmAGO2a         -----VFPGTVVDTKVVHPFEFDFYLCSHYGSLGTSKPTHYHVLWDEHKFNSDDLQKLIY
MtAGO2a         -----VFPGTVVDTKVVHPFEFDFYLCSHYGSLGTSKPTHYHVLWDEHRFTSDNLQKLIY
LjAGO2b         -----ILPGTIVDTKVTHPFEFDFYLCSYYGSLGTSKPTHYHVLWDEHKFKSDELQKLIY
GmAGO2b         -----VLPGTVVDTKVIHPYEFDFYLCSYYGNLGTSKPTHYHVLWDEHKFTSDLLQKLIY
MtAGO2b         -----ILPGTVVDTKVTHPFEFDFYLCSYYGSLGTSKPTHYHVLWDEHKFTSDELQKLIY
LjAGO7          FLYENIPPGTVVDSVITHPKEFDFYLCSHWGVKGTSRPTHYHVLWDENQFTSDELQKLVY
GmAGO7          FLYENIPPGTVVDSVITHPKEFDFYLCSHWGVKGTSRPTHYHVLWDENQFTSDELQKLVY
MtAGO7          FQYENIPPGTVVDSVITHPKEFDFYLCSHWGVKGTSRPTHYHVLLDENKFTSDELQKLVY
GmAGO12b        ------------------------------------RPAHYHVLWDENNFTADEIQSLTN
GmAGO12c        ------------------------------------------------------------
GmAGO12a        ----NILPGTVVDSKICHPTEFDFYLCSHAGIQGTSRPAHYHVLWDENNFTADEIQSLTN
GmAGO12d        ----NILPGTVVDSKICHPSEFDFYLCSHAGIQGTSRPAHYHVLWDENNFTADEIQSLTN
MtAGO12a/b      ----NILPGTVVDTKICHPTEFDFYLCSHAGVQGTSKPAHYHVIWDDNKFSADEIQSLTN
GmAGO10b        ----NILPGTVVDSKICHPTEFDFYLCSHAGIQGTSRPAHYHVLWDENNFTADGIQSLTN
GmAGO10c        ----NILPGTVVDSKICHPTEFDFYLCSHAGIQGTSRPAHYHVLWDENNFTADGIQSLTN
GmAGO10a        ----NILPGTVVDTKICHPTEFDFYLCSHAGIQGTSRPAHYHVLWDENNFTPDGIQSLTN
GmAGO10d        ----NILPGTVVDSKICHPTEFDFYLCSHAGIQGTSRPAHYHVLWDENNFTADGIQSLTN
LjAGO10         ----NIMPGTVVDSKICHPTEFDFYLCSHAGIQGTSRPAHYHVLWDENNFTADGIQSLTN
GmAGO1a         ----NILPGTVVDSKICHPTEFDFYLCSHAGIQGTSRPAHYHVLWDENNFTADALQTLTN
GmAGO1b         ----NILPGTVVDSKICHPTEFDFYLCSHAGIQGTSRPAHYHVLWDENNFTADALQTLTN
LjAGO1          ----NILPGTVVDSKICHPTEFDFYLCSHAGIQGTSRPAHYHVLWDENNFTADGLQSLTN
GmAGO5          ----NIMPGTVVDTHICHPREFDFYLNSHAGMQGTSRPTHYHVLFDENNFTADGLQMFTN
GmAGO5b         ----NILPGTVVDTQICHPREFDFYLNSHAGIQGTSRPTHYHVLFDEITSLLTSCKCLQ-
LjAGO5          ----NIMPGTVVDTSICHPREFDFYLNSHAGIQGTSRPTHYHVLYDENNFTADELQGLTN
LjAGO4a         GSPDNVPPGTIIDNKIGHPRNYDFYLCAHAGMIGTSRPTHYHVLLDQAGFSPDELQELVH
GmAGO4b         GSPDNVPPGTVIDNKICHPRNYDFYLCAHAGMIGTSRPTHYHVLLDQVGFSPDQLQELVH
MtAGO4a         NSPDNVPPGTVIDNKICHPRNYDFYLCAHAGMIGTSRPTHYHVLLDEIGFSPDELQELVH
MtAGO4b         GSPDNVPPGTVVDNKICHPRNYDFYMCAHAGMIGTSRPTHYHVLLDEIGFSPDDLQELVH
MtAGO11b        GSPDNVPPGTVVDNKICHPRNYDFYMCAHAGMIGTSRPTHYHVLLDEIGFSPDDLQELVH
MtAGO4c         RSPDNVPPGTVVDSKICHPRNYDFYMCAHAGMIGTSRPTHYHVLLDEIGFSPDDLQELVH
GmAGO4c         GAPDNVPPGTVIDNKICHPRNYDFYMCAHAGMIGTSRPTHYHVLLDEIGFSPDDLQELVH
GmAGO4a         GAPDNVPPGTVIDNKICHPRNYDFYMCAHAGMIGTSRPTHYHVLLDEIGFSPDDLQELVH
LjAGO4b         GSPDNVPPGTVIDNKICHPRNNDFYMCAHAGMIGTSRPTHYHVLLDDIGFSPDELQELVH
MtAGO4d         ENRNNVPPGTVVDNKICHPRNYDFYMCSHAGRIGTTRPTHYHVLLDEIGFSPDELQEFVH
GmAGO9          RDQTNVPPGTVIDNTVCHPKNNDFYLCAQAGMIGTTRPTHYHVLHDEIGFSADEVQELVH
MtAGO11a        SPQENVSPGTVIDNTICHPKDNDFYMCAHAGRIGTSRPTHYHVLYDEIGFSADNLQEFVH
LjAGO6          SVDN-VPPGTVVDTKIVHPRNYDFYLCAHAGMIGTSRPVHYNVLLDEIGFSPDSLQNLIH
```

```
GmAGO6          GPEN-VPPGTVVDTTITHPRNYDFYMCAHAGMLGTSRPVHYHVLLDEIGFSADGLQNLIH
MtAGO6          ALEKNVPPGTVVDTNIVHPRNYDFYMCAHAGMIGTSRPVHYHVLLDEIGFSSDGLQNLIN


LjAGO2a         DMCFTFARCTKPVSLVPPVYYADLAAYRGRLYYE
GmAGO2a         DMCFTFARCTKPVSLVPPVYYADLTAYRGRLYYE
MtAGO2a         DMCFTFARCTKPVSLVPPVYYADLAAYRGRLYYE
LjAGO2b         EMCFTFARCTKPVSLVPPVYYADLAAYRGRLYH-
GmAGO2b         EMCFTFAKCTKPVSLVPPVYYADLAAYRGRLYH-
MtAGO2b         EMCFTFARCTKPVSLVPPVYYADLAAYRGRLYH-
LjAGO7          NLCYTFVRCTKPISLVPPAYYAHLAAYRGRLYLE
GmAGO7          NLCYTFVRCTKPISLVPPAYYAHLAAYRGRLYLE
MtAGO7          NLCFTFVRCTKPISLVPPAYYAHLAAYRGRLYLE
GmAGO12b        NLCYT-----------------------------
GmAGO12c        ----------------------------------
GmAGO12a        NLCYTYARCTRSVSVVPPAYYAHLAAYRARFYME
GmAGO12d        NLCYTYARCTRSVSVVPPAYYAHLAAYRARFYME
MtAGO12a/b      NLCYTYARCTRSVSLVPPAYYAHLAAYRARFYME
GmAGO10b        NLCYTYARCTRSVSVVPPAYYAHLAAFRARFYME
GmAGO10c        NLCYTYARCTRSVSVVPPAYYAHLAAFRARFYME
GmAGO10a        NLCYTYARCTRSVSVVPPAYYAHLAAFRARFYME
GmAGO10d        NLCYTYARCTRSVSVVPPAYYAHLAAFRARFYME
LjAGO10         NLCYTYARCTRSVSVVPPAYYAHLAAFRARFYME
GmAGO1a         NLCYTYARCTR-SVIVPPAYYAHLAAFRARFYME
GmAGO1b         NLCYTYARCTRSVSIVPPAYYAHLAAFRARFYME
LjAGO1          NLCYTYARCTRSVSIVPPAYYAHLAAFRARFYME
GmAGO5          NLCYTYARCTRSVSIVPPVYYAHLAAFRARCYIE
GmAGO5b         ----------------------------------
LjAGO5          NLCYTYARCTRSVSIVPPAYYAHLAAFRARSYIE
LjAGO4a         SLSYVYQRSTTAISVVAPICYAHLAATQLGQFMK
GmAGO4b         SLSYVYQRSTTAISVVAPICYAHLAATQLGQFMK
MtAGO4a         SLSYVYQRSTTAISVVAPICYAHLAATQLGQFMK
MtAGO4b         SLSYVYQRSTTAISVVAPICYAHLAASQVGQFMK
MtAGO11b        SLSYVYQRSTTAISVVAPICYAHLAASQVGQFMK
MtAGO4c         SLSYVYQRSTTAISVVAPICYAHLAASQVGQFMK
GmAGO4c         SLSYVYQRSTTAISVVAPICYAHLAATQMGQFMK
GmAGO4a         SLSYVYQRSTTAISVVAPICYAHLAATQMGQFMK
LjAGO4b         SLSYVYQRSTTAISVVAPICYAHLAATQIGQFMK
MtAGO4d         SLSYVYQRSTTAVSVVAPICYAHLAASQVAQFMK
GmAGO9          SLSYTYQRSTTAVSLVAPICYAHLAAAQMAQFMK
MtAGO11a        SLCYVHQRSTNAISIVAPIYYADLAAAQIAQFIK
LjAGO6          SLSYANQRSSIATSIVAPIHYAHHAAAQMRQVLN
GmAGO6          SLSYVNQRSTIATSVVAPICYAHHAAAQMGQLLN
MtAGO6          SLSYVNQRSTAATSIVAPIYYAHHAAAQMRKFMN
```

Data Sheet 5. Sequence comparison of PIWI domains of AGO proteins from *M. truncatula*, *L. japonicus* and *G. max*. Sequence alignement was done thanks to T-Coffee software. PIWI domain of AGO protein sequences in the three model legumes were obtained as described by Capitao et al., 2011. Conserved amino acid residues cited in the text are colored.
